# Transcription bodies regulate gene expression by sequestering CDK9

**DOI:** 10.1038/s41556-024-01389-9

**Published:** 2024-04-08

**Authors:** Martino Ugolini, Maciej A. Kerlin, Ksenia Kuznetsova, Haruka Oda, Hiroshi Kimura, Nadine L. Vastenhouw

**Affiliations:** 1 Center for Integrative Genomics (CIG), University of Lausanne (UNIL), Lausanne, Switzerland; 2 Max Planck Institute of Molecular Cell Biology and Genetics (MPI-CBG), Dresden, Germany; 3 Cell Biology Center, Institute of Innovative Research, Tokyo Institute of Technology, Yokohama, Japan; 4 Present Address: Institute of Human Genetics, CNRS, Montpellier, France

**Keywords:** Nuclear organization, Transcription

## Abstract

The localization of transcriptional activity in specialized transcription bodies is a hallmark of gene expression in eukaryotic cells. It remains unclear, however, if and how transcription bodies affect gene expression. Here we disrupted the formation of two prominent endogenous transcription bodies that mark the onset of zygotic transcription in zebrafish embryos and analysed the effect on gene expression using enriched SLAM-seq and live-cell imaging. We find that the disruption of transcription bodies results in the misregulation of hundreds of genes. Here we focus on genes that are upregulated. These genes have accessible chromatin and are poised to be transcribed in the presence of the two transcription bodies, but they do not go into elongation. Live-cell imaging shows that disruption of the two large transcription bodies enables these poised genes to be transcribed in ectopic transcription bodies, suggesting that the large transcription bodies sequester a pause release factor. Supporting this hypothesis, we find that CDK9—the kinase that releases paused polymerase II—is highly enriched in the two large transcription bodies. Overexpression of CDK9 in wild-type embryos results in the formation of ectopic transcription bodies and thus phenocopies the removal of the two large transcription bodies. Taken together, our results show that transcription bodies regulate transcription by sequestering machinery, thereby preventing genes elsewhere in the nucleus from being transcribed.

## Abstract (web summary)

Ugolini et al. show that transcription bodies regulate gene expression during zygotic genome activation in zebrafish development by sequestering CDK9 to limit the transcription of genes away from transcription bodies.

## Main

**Fig. 1:**
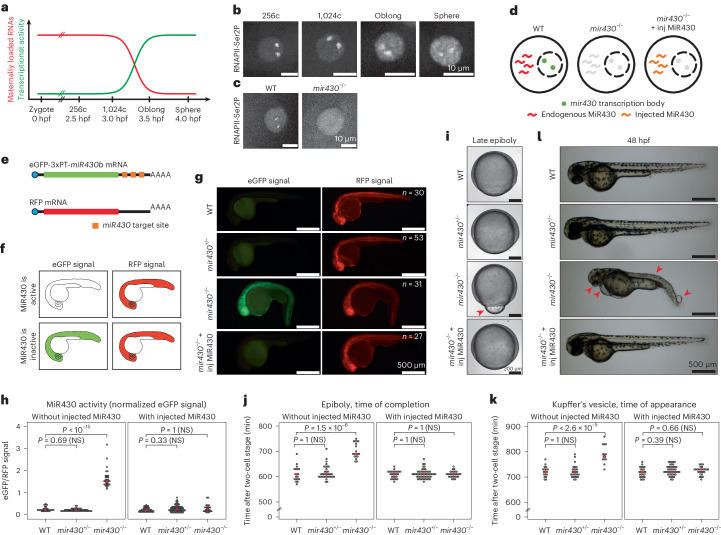
Disruption of *mir430* transcription bodies does not substantially impact development. **a**, Schematic representation of the maternal-to-zygotic transition in zebrafish embryos. **b**,**c**, Visualization of elongating RNAPII (Ser2P) with Fabs in WT embryos at the 256-cell (256c), 1,024-cell (1,024c), Oblong and Sphere stages (**b**) and in WT and *mir430*^−/−^ embryos at the 128-cell stage (**c**). Shown are representative micrographs of individual nuclei, extracted from a spinning disk confocal microscopy timelapse. **d**, Schematic representation of a nucleus in WT, *mir430*^−/−^ and *mir430*^−/−^ with injected MiR430 embryos. **e**, Approach to assess rescue of *miR430* activity, as previously described^24^. The expression of eGFP encoded by an mRNA with three perfect target sites for *miR430* is compared with the expression of RFP encoded by an mRNA without such sites (Methods). **f**, Schematic representation of expected eGFP and RFP expression in embryos with active and inactive MiR430 microRNA activity. **g**, Representative micrographs showing eGFP and RFP expression in WT, *mir430*^+/−^, *mir430*^−/−^ and *mir430*^−/^ with injected MiR430 embryos at 24 hpf. *N* = 3 biologically independent experiments. **h**, Rescue of *miR430* activity was assessed in different genotypes at 24 hpf. Normalized eGFP signal in WT, *mir430*^+/−^ and *mir430*^−/−^ embryos without (left) and with (right) injected MiR430. *N* = 3 biologically independent experiments, *n* = 30 (WT without injected MiR430), *n* = 53 (*mir430*^+^^/−^ without injected MiR430), *n* = 31 (*mir430*^−/−^ without injected MiR430), *n* = 45 (WT with injected MiR430), *n* = 74 (*mir430*^+/−^ with injected MiR430), *n* = 27 (*mir430*^−/−^ with injected MiR430). Kruskal–Wallis tests were performed (without injected MiR430: *χ*^2^ = 67.892, d.f. = 2, *P* value = 1.809 × 10^−15^; with injected MiR430: *χ*^2^ = 2.5872, d.f. = 2, *P* value = 0.2743). When this test was statistically significant (*P* value < 0.05), pairwise comparisons with Bonferroni correction were performed using a pairwise Wilcoxon rank-sum test. A comparison was considered significant when adjusted *P* value was <0.05, and adjusted *P* values were reported using WT as reference. **i**, Rescue of *miR430* activity as assessed by epiboly progression. Shown are representative micrographs of embryos at late epiboly stage in different genotypes. The misregulation of yolk internalization in *mir430*^−/−^ embryos is indicated. **j**, Time at which epiboly is completed in different genotypes without (left) and with (right) injected MiR430 RNA. *N* = 3 (without injected MiR430) and *N* = 4 (with injected MiR430) biologically independent experiments, *n* = 19 (WT without injected MiR430), *n* = 34 (*mir430*^+/−^ without injected MiR430), *n* = 19 (*mir430*^−/−^ without injected MiR430), *n* = 27 (WT with injected MiR430), *n* = 53 (*mir430*^+/−^ with injected MiR430), *n* = 28 (*mir430*^−/−^ with injected MiR430). Kruskal–Wallis tests were performed (without injected MiR430: *χ*^2^ = 38.379, d.f. = 2, *P* value = 4.635 × 10^−9^; with injected MiR430: *χ*^2^ = 0.00080597, d.f. = 2, *P* value = 0.9996). When this test was statistically significant (*P* value < 0.05), pairwise comparisons with Bonferroni correction were performed using a pairwise Wilcoxon rank-sum test. A comparison was considered significant when adjusted *P* value was <0.05, and adjusted *P* values were reported using WT as reference. **k**, Time at which Kupffer’s vesicle appears in different genotypes without (left) and with (right) injected MiR430. *N* = 3 (without injected MiR430) and *N* = 4 (with injected MiR430) biologically independent experiments, *n* = 18 (WT without injected MiR430), *n* = 27 (*mir430*^+/−^ without injected MiR430), *n* = 14 (*mir430*^−/−^ without injected MiR430), *n* = 25 (WT with injected MiR430), *n* = 44 (*mir430*^+/−^ with injected MiR430), *n* = 26 (*mir430*^−/−^ with injected MiR430). Kruskal–Wallis tests were performed (without injected MiR430: *χ*^2^ = 26.038, d.f. = 2, *P* value = 2.218 × 10^−6^; with injected MiR430: *χ*^2^ = 2.543, d.f. = 2, *P* value = 0.2804). When this test was statistically significant (*P* value < 0.05), pairwise comparisons with Bonferroni correction were performed using a pairwise Wilcoxon rank-sum test. A comparison was considered significant when adjusted *P* value was <0.05, and adjusted *P* values were reported using WT as reference. **l**, Larvae at 48 hpf for different genotypes. Representative micrographs are shown. The malformation of trunk morphology and eye, the development of heart oedema, and the appearance of blisters at the tail tip in *mir430*^−/−^ embryos are indicated (red arrowheads). Source numerical data are available in Source data. Source data

**Extended Data Fig. 1:**
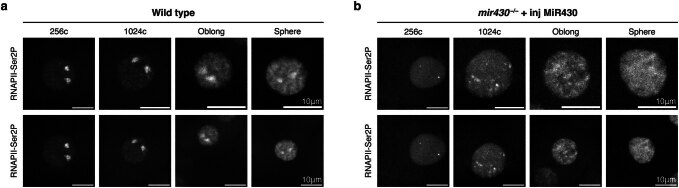
Changes in elongating RNAPII signal during ZGA in WT and *mir430*^-/-^ + inj MiR430 nuclei. **a,b**. Visualization of elongating RNAPII (Ser2P) with antigen-binding fragments (Fabs) in WT (**a**) and *mir430*^-/-^ + inj MiR430 (**b**) embryos. Shown are representative micrographs of individual nuclei at 256-cell, 1024-cell, Oblong and Sphere stage, extracted from a spinning disk confocal microscopy timelapse. Nuclei are shown at increasing scale (top) and constant scale (bottom). N = 3 biologically independent experiments for each genotype.

RNA polymerase II (RNAPII) and transcription factors are often concentrated in specialized transcription bodies^1–10^, and it has been suggested that these can bring together several genes^11–14^. This has led to the hypothesis that transcription bodies increase the efficiency of transcription by promoting the biomolecular interactions underlying gene expression^15–17^. Analysis of gene expression in artificially induced condensates supports this model^18^. To understand how transcription bodies affect gene expression in vivo, however, it is necessary to analyse endogenous transcription bodies. This has been difficult because transcription bodies are often small, short-lived and numerous. Here, we use the onset of transcription in zebrafish embryos to overcome this problem. In zebrafish embryos, development is driven initially by maternally loaded RNA and protein (Fig. 1a)^19^. Transcription begins gradually during a process called zygotic genome activation (ZGA). Before transcriptional activity can be seen throughout the nucleus, it is confined to two micron-sized transcription bodies (Fig. 1b and Extended Data Fig. 1)^9,20–23^, nucleated by the *mir430* locus, which contains several copies of the *mir430* gene^21,24^. These bodies are enriched for RNAPII serine 5 and serine 2 phosphorylation (Ser5P, Ser2P), which mark the initiating and elongating form of RNAPII, respectively, as well as the transcription factors Nanog and Sox19b^9,20–22^.

## Results

### Loss of *mir430* transcription bodies does not substantially impact development

**Extended Data Fig. 2:**
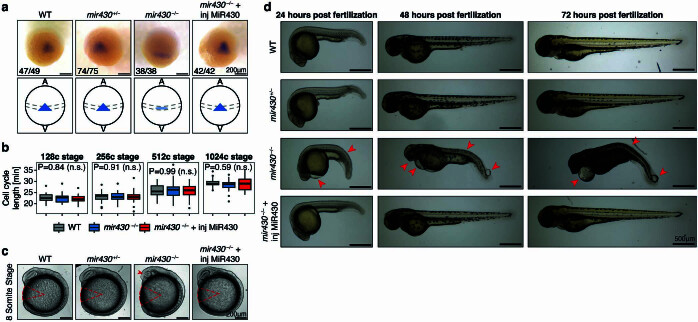
Phenotypic analysis of *mir430*^-/-^ embryos with and without injected MiR430. **a**. Whole mount *in situ* hybridization (WM-ISH) of *Goosecoid (Gsc)* in WT, *mir430*^+/-^, *mir430*^-/-^ and *mir430*^-/-^ + inj MiR430 embryos at Shield stage. Representative micrographs show the embryos in dorsal view. Misexpression (reduced expression levels and reduced extension of expression pattern) of *Gsc* can be observed in *mir430*^-/-^ embryos. The fraction in the lower left corner represents the frequency with which the shown phenotype was observed. A schematic representation of the phenotypes is shown below. N = 3 biologically independent experiments. **b**. Duration of the cell cycle at 128-, 256-, 512-, and 1024-cell stage in WT, *mir430*^+/-^, *mir430*^-/-^ and *mir430*^-/-^ + inj MiR430 embryos. Measurement based on live bright-field imaging with high temporal resolution (2 frames per minute). N = 3 biologically independent experiments, with n = 29 (WT), 26 (*mir430*^-/-^), and 27 embryos (*mir430*^-/-^ + inj MiR430). Boxplots show median, quartiles, minimum and maximum and 1.5x interquartile range. Individual points represent outliers. At each stage, Kruskal-Wallis tests were performed (128c: ***χ***^2^ = 0.34364, df = 2, P-value = 0.8421, 256c: ***χ***^2^ = 0.18903, df = 2, P-value = 0.9098, 512c: ***χ***^2^ = 0.016565, df = 2, P-value = 0.9918, 1024c: ***χ***^2^ = 1.0398, df = 2, P-value = 0.5946), none of which was significant (P-value < 0.05). See Methods for a detailed description of the quantification. **c**. Embryos at 8-Somite stage for WT, *mir430*^+/-^, *mir430*^-/-^ and *mir430*^-/-^ + inj MiR430 embryos. The reduced body axis extension and the malformation of the optic primordium in *mir430*^-/-^ embryos are highlighted. Representative micrographs are shown. N = 3 biologically independent experiments. **d**. Larvae at 24, 48 and 72 hours post fertilization (hpf) for WT, *mir430*^+/-^, *mir430*^-/-^ and *mir430*^-/-^ + inj MiR430 embryos. The malformation of trunk morphology and of the eye, the development of heart oedema and the appearance of blisters at the tail tip in *mir430*^-/-^ embryos are highlighted. Representative micrographs are shown. N = 3 biologically independent experiments. The 48hpf stage is also shown in Fig. 1l. Source numerical data are available in source data. Source data

To investigate the role of transcription bodies in transcription, we generated a fish line in which the *mir430* locus is deleted^9^, resulting in the specific disruption of the two *mir430* transcription bodies, as observed by the loss of Ser2P signal (Fig. 1c). *miR430* is required for downregulating maternally loaded transcripts and its absence results in severe developmental phenotypes^24,25^. Thus, effects on gene expression and development in the *mir430* mutant may be caused by the loss of the *mir430* transcription bodies, or the loss of the mature microRNA MiR430. To uncouple these two components and allow us to focus on the former, we supplemented mutant embryos with the mature form of microRNA MiR430 (Fig. 1d)^24^, which restored degradation of mRNAs containing MiR430 binding sites (Fig. 1e–h). This rescued the delay in epiboly (Fig. 1i,j) and the aberrant expression pattern of *goosecoid* (Extended Data Fig. 2a), which are characteristic for the loss of MiR430 (refs. ^24,25^). Embryos supplemented with mature microRNAs appeared normal, with embryonic cell cycle length (Extended Data Fig. 2b), the appearance of the Kupffer’s vesicle (Fig. 1k) and overall developmental progression (Fig. 1l and Extended Data Fig. 2c,d) being indistinguishable from wild-type (WT) and heterozygous siblings; embryos developed into fertile fish. Thus, the disruption of two large transcription bodies does not result in obvious developmental defects, and we can use it to analyse the effect of transcription bodies on gene expression.

### Loss of *mir430* transcription bodies causes widespread misregulation of gene expression

**Fig. 2:**
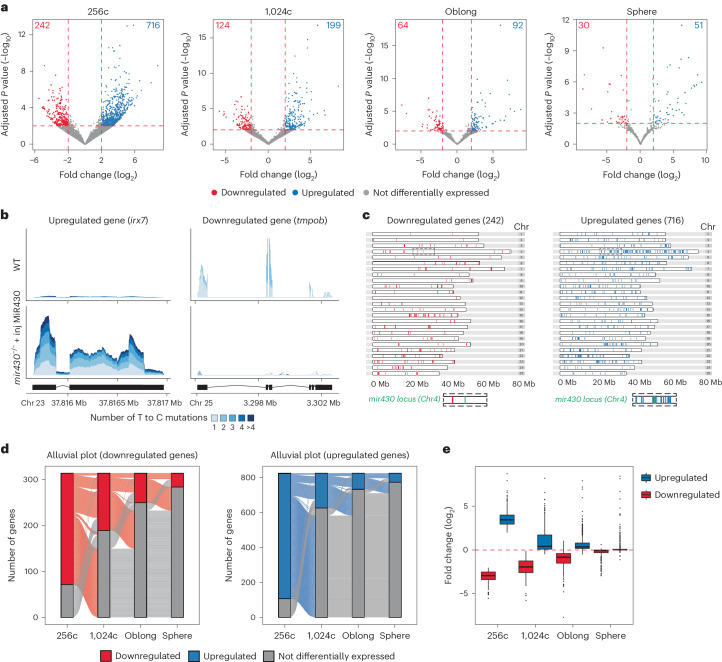
Disruption of *mir430* transcription bodies causes widespread misregulation of gene expression that recovers over time. **a**, Volcano plots showing upregulated and downregulated genes in *mir430*^−/− ^+ inj MiR430 (*n* = 3 biologically independent samples) versus WT (*n* = 3 biologically independent samples) embryos at 256-cell, 1,024-cell, Oblong and Sphere stages. Wald test with Benjamini–Hochberg correction was performed, and genes with adjusted *P* values <0.01 were considered significantly differentially expressed. Genes whose coverage is shown in **b** are shown in black in the left-most plot. **b**, Coverage plot of *irx7* (upregulated) and *tmpob* (downregulated) in WT and *mir430*^−/−^ + inj MiR430. The single strata visualize the labelling degree of the reads. **c**, Distribution of upregulated and downregulated genes across the genome. The *mir430* locus on chromosome (Chr) 4 is shown in green and an expansion of its surrounding sequence is shown at the bottom of the panel. **d**, Alluvial plots showing the overlap between downregulated (left) and upregulated (right) genes at different stages of ZGA. **e**, Difference in average gene expression between *mir430*^−/−^ + inj MiR430 and WT embryos across stages for those genes that were identified to be differentially expressed at 256-cell stage (*n* = 242 downregulated genes and *n* = 716 upregulated genes). Boxplots show median, quartiles, minimum and maximum, and 1.5× interquartile range. Individual points represent outliers. Source numerical data are available on GEO (GSE248237).

**Extended Data Fig. 3:**
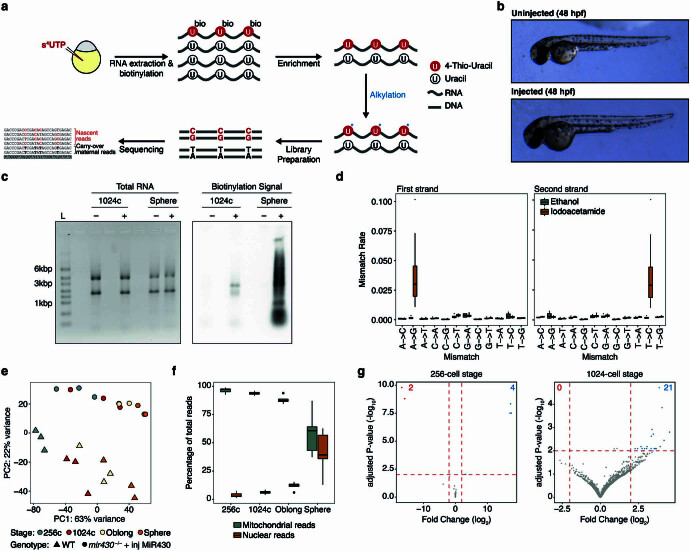
Establishment of enriched SLAM-Sequencing (eSLAM-Seq) in zebrafish embryos. **a**. Schematic representation of eSLAM-Seq protocol. See Methods for more detail. **b**. Representative micrographs of larvae that were/were not injected with 50 nM/embryo of s^4^UTP at the 1-cell stage. No developmental malformations were observed upon injection. **c**. Equal amounts of biotinylated and heat-denatured total RNA derived from injected (+) and uninjected (-) embryos at 1024-cell and Sphere stage were run on an agarose gel (left panel). The RNA was then transferred on a nylon membrane and biotinylation detected by Streptavidin-HRP (right panel). Biotinylation was only observed in RNA derived from injected embryos, showing that biotinylation is specific for s^4^UTP-labeled RNA. N = 2 biologically independent experiments. L: RNA Ladder. **d**. Box plot representation of mismatch rate in Iodoacetamide- (N = 24) and Ethanol-treated (N = 4) samples. As expected, only Iodoacetamide-treated samples show a high level in A > G (First strand) and T > C (Second strand) mismatch rate. Boxplots show median, quartiles, minimum and maximum, and 1.5x interquartile range. Individual points represent outliers. **e**. Principal Component Analysis (PCA) plot based on the top 500 genes (selected by highest variance in expression levels). Samples separate according to their developmental stage along the first PC and according to their experimental condition (WT and *mir430*^-/-^ + inj MiR430) along the second PC. **f**. Box plot representation of percentage of mitochondrial and nuclear reads, separated by developmental stage (N = 6 biologically independent samples). Most reads are of mitochondrial origin at early ZGA, as shown before^35^, with a gradual increase in the nuclear fraction during later stages. Boxplots show median, quartiles, minimum and maximum, and 1.5x interquartile range. Individual points represent outliers. **g**. Volcano plot showing up- and downregulated genes in WT + inj MiR430 (n = 3 biologically independent samples) *vs*. WT embryos (n = 3 biologically independent samples) at 256- and 1024-cell stage. Wald test with Benjamini-Hochberg correction was performed, and genes with adjusted P-values < 0.01 were considered significantly differentially expressed. Source numerical data are available on GEO (GSE248237) and unprocessed blots are available in source data. Source data

**Extended Data Fig. 4:**
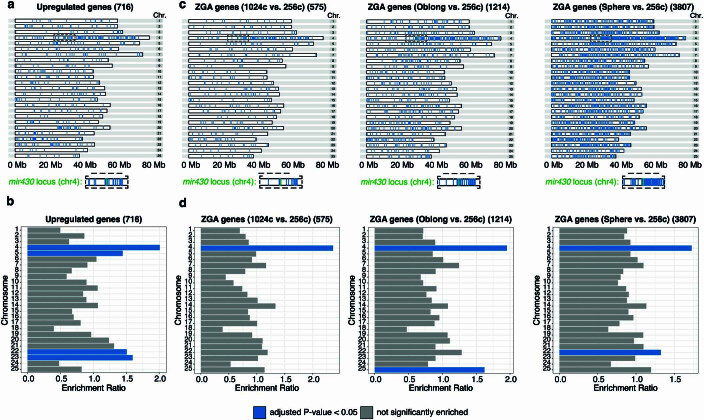
The genomic distribution of upregulated genes is similar to that of ZGA genes. **a**. Distribution of upregulated genes (*mir430*^-/-^ + inj MiR430 *vs*. WT at 256c) across the genome (as in Fig. 2c). The *mir430* locus on chromosome 4 is shown in green and a zoom of its surrounding area is shown at the bottom of the panel. **b**. The enrichment (Observed vs. Expected) of genes shown in (**a**) on each chromosome. Significant enrichment was calculated using the hypergeometric distribution, and P-values were corrected for multiple testing with the Benjamini-Hochberg method. Significant enrichment (adjusted P-value < 0.05) is shown as blue bars. **c**. Distribution of ZGA genes (three classes: upregulated in WT 1024c *vs*. 256c, WT Oblong *vs*. 256c and WT Sphere *vs*. 256c) across the genome. **d**. The enrichment (Observed vs. Expected) of genes shown in (**c**) on each chromosome. Significant enrichment was estimated using the hypergeometric distribution, and P-values were corrected for multiple testing with the Benjamini-Hochberg method. Significant enrichment (adjusted P-value < 0.05) is shown as blue bars. Source numerical data are available on GEO (GSE248237).

To assess the effect of the specific disruption of *mir430* transcription bodies on transcriptional activity, we developed enriched thiol(SH)-linked alkylation for the metabolic sequencing of RNA (eSLAM-seq), which uses a combination of protocols to label, enrich and detect nascent transcripts (Extended Data Fig. 3a–f and Methods), because the large amounts of maternally loaded RNA in early embryos (Fig. 1a) mask nascent RNAs in total RNA sequencing approaches. We first used eSLAM-seq to test whether the injection of the mature form of microRNA MiR430 in one-cell stage embryos affects zygotic transcription, and found that only very few genes are affected (Extended Data Fig. 3g). Next, we compared gene expression between WT and *mir430*^−/−^ + injected (inj) MiR430 embryos at the 256-cell stage, when transcriptional activity is restricted largely to *mir430* transcription bodies in WT embryos (Fig. 1b). We observed an important number of genes (242) that were downregulated in the absence of *mir430* transcription bodies and an even higher number of genes (716) that were upregulated (Fig. 2a,b and Supplementary Table 1). Plotting the genomic location of genes that were upregulated and downregulated showed that both classes of genes are distributed across the genome (Fig. 2c and Extended Data Fig. 4). We conclude that the absence of two large transcription bodies has a widespread effect on transcription.

### Misregulation of gene expression is short lived

The identification of hundreds of upregulated and downregulated genes in *mir430*^−/−^ + inj MiR430 versus WT embryos at the 256-cell stage contrasts with the observation that these embryos develop normally (Fig. 1). To reconcile these two observations, we generated eSLAM-seq data for WT and *mir430*^−/−^ + inj MiR430 embryos at the 1,024-cell, Oblong and Sphere stages and compared it with data obtained at the 256-cell stage. Overall, we find that fewer genes are misregulated at later stages, from 716 upregulated and 242 downregulated at 256-cell to 51 upregulated and 30 downregulated at the Sphere stage (Fig. 2a). The misregulated genes at later stages are—to a large extent—a subset of the misregulated genes at 256-cell stage (Fig. 2d), suggesting that gene expression recovers over time. Indeed, when we focus on the expression levels of genes that are misregulated in *mir430*^−/− ^+ inj MiR430 embryos at the 256-cell stage (with a median of 3.5 log_2_ fold difference with WT for the upregulated genes, and 2.9 log_2_ fold difference for the downregulated genes; Fig. 2e), we find that they recover gradually during consecutive developmental stages, reaching WT expression levels at Sphere stage (with a median of 0.03 log_2_ fold difference with WT for the upregulated genes, and 0.07 log_2_ fold difference for the downregulated genes; Fig. 2e). We conclude that, in the absence of *mir430* transcription bodies, gene expression is misregulated, but that this effect is short lived as expression levels return to WT levels in a timeframe of a couple of cell cycles. This probably explains the lack of obvious developmental defects in *mir430*^−/−^ + inj MiR430 embryos.

### Downregulated genes do not consistently localize to *mir430* transcription bodies

**Fig. 3:**
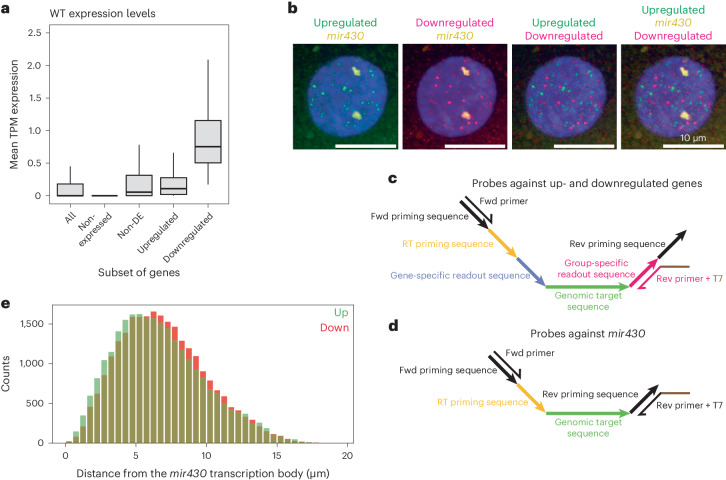
Characterization of downregulated genes. **a**, Average expression level in transcripts per million (TPM) in WT embryos for all genes (*n* = 32,428), non-expressed genes (*n* = 9,545), non-differentially expressed (DE) genes (*n* = 19,426) and upregulated (*n* = 716) and downregulated (*n* = 242) genes (*mir430*^−/−^ + inj MiR430 versus WT at 256-cell stage). Boxplots show median, quartiles, minimum and maximum, and 1.5× interquartile range. Outliers are not shown. **b**, Representative images of a DNA-FISH experiment for upregulated genes (green), downregulated genes (magenta) and *mir430* (yellow) in a nucleus of a WT embryo at the 256-cell stage. *N* = 3. Nuclei were also stained with DAPI (blue). Images shown are maximum intensity projections. **c**,**d**, Schematic representation of oligopaint probe design for upregulated and downregulated genes (**c**) and the *mir430* locus (**d**). Primers used for qPCR amplification and their complementarity within a probe are shown. Probe sequences are reported in Supplementary Table 2. Fwd, forward; Rev, reverse. **e**, Distributions of 3D distances of upregulated (up) genes from the *mir430* locus (green) and downregulated (down) genes from the *mir430* locus (red) in WT embryos. Quantification of one biological replicate was performed. *N* = 19 embryos, *n* = 643 nuclei. Source numerical data are available on GEO (GSE248237).

We then further analysed genes that are misregulated at the 256-cell stage. The downregulated genes are genes that—in WT embryos—are expressed at 256-cell stage (Fig. 3a). Their identification shows that transcription bodies facilitate transcription of genes other than *mir430* itself. It has been proposed that transcription bodies may do so by bringing or keeping genes together in nuclear space and increasing transcriptional efficiency^11–14^. In DNA fluorescence in situ hybridization (DNA-FISH) experiments, however, we were unable to see a consistent colocalization of downregulated genes with the *mir430* transcription bodies, with only 0.14% of spots that detect downregulated genes localizing within 0.5 μm of *mir430* transcription bodies (Fig. 3b–e and Methods). It is thus unclear how transcription bodies positively impact transcription of genes in trans and more work will be required to investigate this.

### Loss of *mir430* transcription bodies causes premature transcription activation

**Fig. 4:**
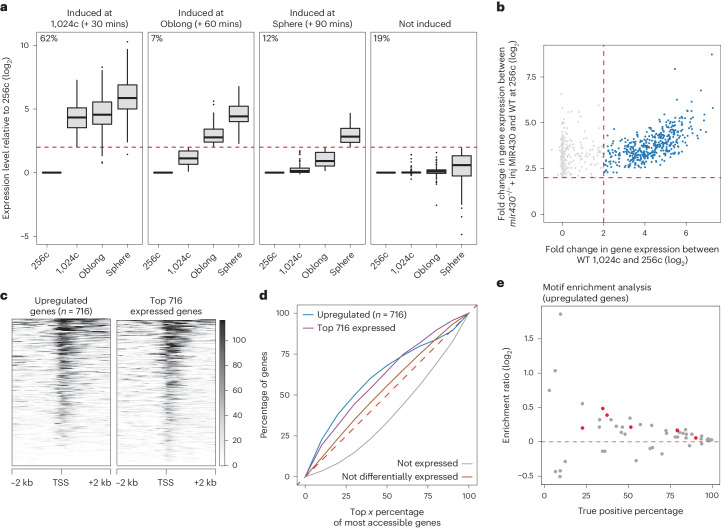
Loss of *mir430* transcription bodies causes premature gene activation. **a**, Time of induction in WT embryos for the 716 genes that are upregulated in *mir430*^−/−^ + inj MiR430 versus WT embryos (at 256-cell stage). Expression at 256-cell stage is used as a reference. The genes are split into four groups based on when they are induced (*n* = 446 induced genes at 1,024-cell stage; *n* = 51 induced genes at Oblong; *n* = 83 genes induced at Sphere; *n* = 136 not induced). The percentage of genes in each group is indicated. Boxplots show median, quartiles, minimum and maximum, and 1.5× interquartile range. Individual points represent outliers. **b**, Scatterplot representing the fold change in expression between *mir430*^−/−^ + inj MiR430 and WT embryos at 256-cell stage on the *y* axis, and between 1,024-cell stage and 256-cell stage in WT embryos on the *x* axis. All upregulated genes are shown in grey, and genes induced at 1,024-cell stage (62%) are shown in blue. **c**, Heatmap of chromatin accessibility at 256-cell stage of the 716 genes that are upregulated in *mir430*^−/−^ + inj MiR430 embryos compared with WT embryos at the 256-cell stage, and—for comparison—the 716 most expressed genes in WT embryos at the 256-cell stage. Genes are ranked by accessibility. **d**, Graph showing the fraction of promoters of the 716 upregulated (in *mir430*^−/−^ + inj MiR430 versus WT at the 256-cell stage) and the 716 most expressed genes (in WT at 256-cell stage) amongst the x% most accessible promoters at 256-cell stage in WT embryos. The distribution of non-expressed genes as well as the non-differentially expressed genes (*mir430*^−/−^ + inj MiR430 versus WT at 256-cell stage) are shown for comparison. **e**, Scatterplot representing the enrichment ratio (log_2_) of transcription factor motifs in the promoters (TSS ± 2 kb) of upregulated genes compared with the promoters of not expressed genes (*y* axis) and the percentage of promoters of the upregulated genes that have the motif (*x* axis). Only motifs whose corresponding protein is translated during early development^27^ were considered, and only motifs with a *P* value < 0.05 and E-value ≤ 10 are shown. Motifs corresponding to the three pluripotency factors Nanog, Sox19b and Pou5f3 (POU5F1, Pou5f1, Pou5f1::Sox2, POU5F1B, POU2F1, POU2F1::SOX2) are labelled in red. Source numerical data are available in Source data and on GEO (GSE248237 and GSE130944). Source data

**Extended Data Fig. 5:**
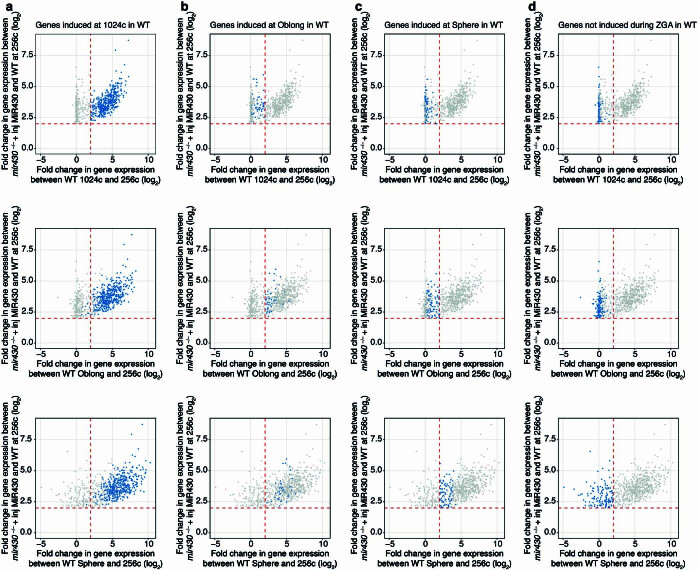
Correlation between expression level in *mir430*^-/-^ + inj MiR430 *vs*. WT and 1024-cell, Oblong and Sphere *vs*. 256-cell in WT embryos. **a**. Scatterplots representing the fold change in gene expression (log_2_ scale) between *mir430*^-/-^ + inj MiR430 and WT at 256-cell (y-axis), and the fold change in gene expression (log_2_ scale) between 1024-cell and 256-cell (left), Oblong and 256-cell (central) and Sphere and 256-cell (right) in WT embryos (x-axis). All upregulated genes are shown in grey, and genes induced at 1024-cell are highlighted in blue. The top graph is also shown in Fig. 4b. **b-d**. Same as in (a) but genes that are highlighted represent genes that are induced at Oblong **(b)**, Sphere **(c)**, or not induced during ZGA **(d)**. Source numerical data are available on GEO (GSE248237).

Here, we focus on the upregulated genes. Their high number suggests that *mir430* transcription bodies sequester components of the transcriptional machinery, preventing genes elsewhere in the nucleus from being transcribed. To investigate this sequestration model, we characterized genes that are upregulated in response to *mir430* transcription body removal. We first asked when these genes are induced in WT embryos. We analysed their expression at the 1,024-cell stage, Oblong and Sphere stages, and compared this with their expression at the 256-cell stage. We found that most of the genes (81%) are strongly activated during genome activation in WT embryos (Fig. 4a), indicating that loss of the *mir430* transcription bodies specifically activates the expression of genes that are about to be activated, with 62% (446 of 716) being advanced by 30 min (at the 1,024-cell stage in WT; Fig. 4a). The specific upregulation of ZGA genes explains the overrepresentation of upregulated genes on Chr4, as ZGA genes are also overrepresented on Chr4 (Extended Data Fig. 4). When focusing on genes that are advanced by 30 min, we observe a good correlation between their degree of upregulation between *mir430*^−/−^ + inj MiR430 and WT at 256-cell stage, and between 1,024-cell and 256-cell stages in WT (Fig. 4b and Extended Data Fig. 5), suggesting that the activation of genes in response to disruption of transcription bodies is similar to their activation during genome activation. We conclude that disruption of transcription bodies specifically activates ZGA genes prematurely.

### Loss of *mir430* transcription bodies facilitates the transition to elongation state

We then investigated the chromatin accessibility state of the genes that are upregulated. We analysed an ATAC-Seq dataset we had previously generated in WT embryos^26^ and found that the transcription start site (TSS) of genes that are upregulated in the *mir430*^−/−^ + inj MiR430 are highly accessible in WT embryos (Fig. 4c). In fact, they are among the highest accessible genes (Fig. 4d) being as accessible as those of the most highly expressed genes (Fig. 4c,d), even though they are barely expressed (Fig. 3a). This suggests that potentially sequestered factor(s) function downstream of transcription factor binding and chromatin opening. Consistent with this, motif analysis of the promoters of upregulated genes did not identify transcription factor motifs that were both sufficiently enriched compared with unexpressed genes and present in a sufficiently high fraction of the upregulated genes to explain our observations (Fig. 4e). Even motifs of Nanog, Pou5f3 and Sox19b—transcription factors shown to be required for the activation of transcription during ZGA in zebrafish^9,26–29^—did not fulfil these two requirements. Taken together, our results suggest that the presence of the *mir430* transcription bodies prevents a substantial number of genes, that are otherwise poised for activation, from going into elongation.

**Fig. 5:**
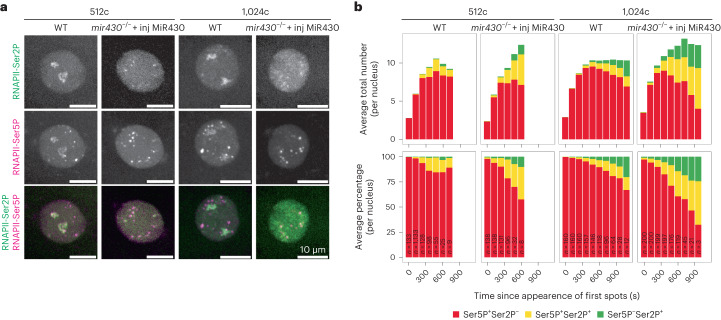
Loss of *mir430* transcription bodies causes ectopic transcription bodies to go into elongation. **a**, Visualization of RNAPII-Ser2P and RNAPII-Ser5P with Fabs in WT and *mir430*^−/−^ + inj MiR430 embryos at 512- and 1,024-cell stages. Shown are representative micrographs of individual nuclei. See Extended Data Fig. 6 for complete cell cycles. *N* = 3 biologically independent experiments. **b**, Quantification of Ser5P-positive/Ser2P-negative (red), Ser5P-positive/Ser2P-positive (yellow) and Ser5P-negative/Ser2P-positive (green) transcription bodies during the cell cycle at 512-cell and 1,024-cell stages as shown in **a**. Number (upper panel) and percentage (lower panel) are shown. *N* = 3 biologically independent experiments, number of nuclei (*n*) analysed at each timepoint, in each cell cycle and in each condition (WT and *mir430*^−/−^ + inj MiR430) are reported. Source numerical data are available in Source data. Source data

**Extended Data Fig. 6:**
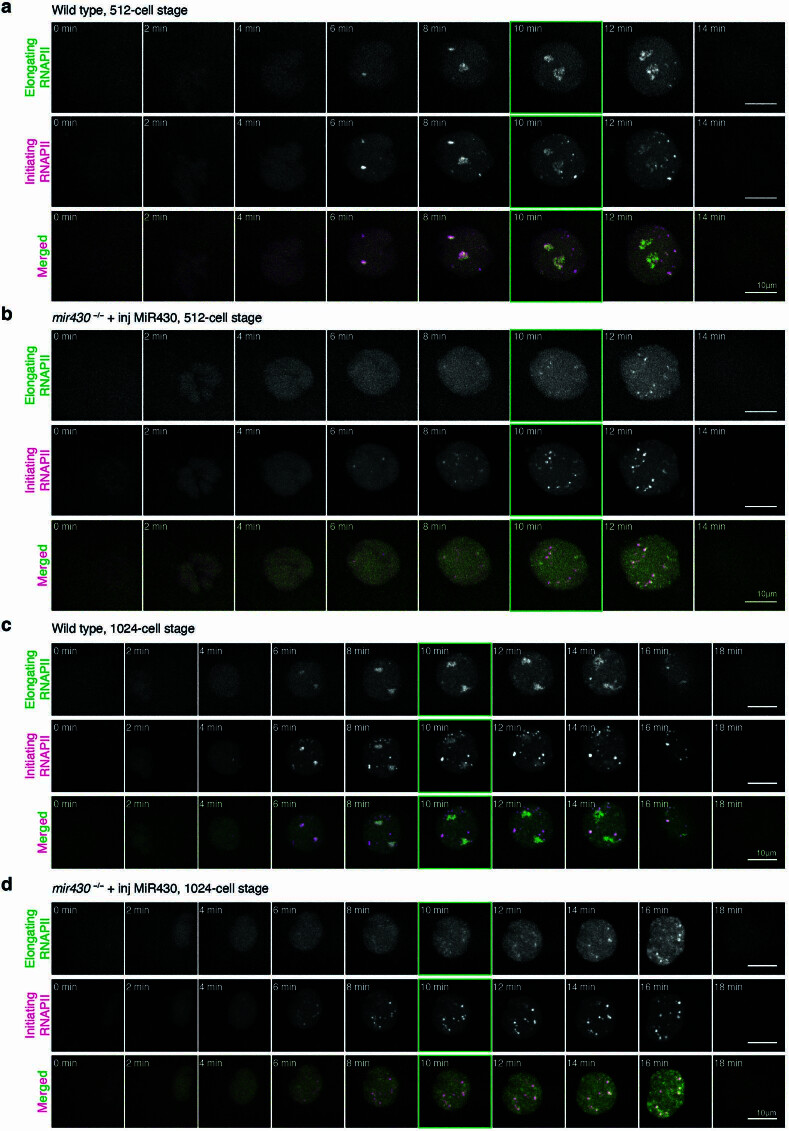
Stalled transcription bodies go into productive elongation in *mir430*^-/-^ + inj MiR430 embryos. Visualization of initiating (Ser5P) and elongating (Ser2P) RNAPII with Fabs in WT **(a, c)** and *mir430*^-/-^ + inj MiR430 **(b, d)** embryos at 512-cell stage **(a, b)** and 1024-cell stage **(c, d)**. Shown are representative images of individual nuclei, extracted from a spinning disk confocal microscopy timelapse. The micrographs shown in the main figure are highlighted in green. N = 3 biologically independent experiments. Information on quantification can be found in the legend of Fig. 5b.

This reminded us of the observation that WT embryos from the 256-cell stage onward, in addition to the *mir430* transcription bodies in which both transcription initiation and elongation can be observed, display transcription bodies that are positive for transcription initiation but not elongation^9^. We were previously unable to explain this observation but now hypothesize that *mir430* transcription bodies might sequester a pause release factor, causing genes in these transcription bodies to be stalled at the initiation phase. If this were true, these transcription bodies would be predicted to acquire elongation signals in nuclei of *mir430*^−/−^ + inj MiR430 embryos. To investigate this, we injected one-cell WT and *mir430*^−/−^ + inj MiR430 embryos with fluorescently labelled antigen-binding fragments (Fabs) recognizing RNAPII-Ser5P (transcription initiation) and RNAPII-Ser2P (transcription elongation) as described previously^9,22,23^. Consistent with our hypothesis, we saw more Ser2P-positive transcription bodies in *mir430*^−/−^ + inj MiR430 than in WT embryos during the 512- and 1,024-cell stages (Fig. 5a and Extended Data Fig. 6). Quantification of initiation-only, initiation/elongation and elongation-only transcription bodies confirmed that more transcription bodies go into productive elongation in *mir430*^−/−^ + inj MiR430 than in WT embryos (with a maximum of 15% elongation-positive transcription bodies in WT versus 33% in *mir430*^−/−^ + inj MiR430 at the 256-cell stage, and a comparable difference at the 1,024-cell stage; Fig. 5b). Thus, removal of *mir430* transcription bodies causes those transcription bodies that are stalled in the initiation phase to transition to the transcription elongation phase.

### CDK9 sequestration in *mir430* transcription bodies inhibits transcription elongation elsewhere in the nucleus

**Fig. 6:**
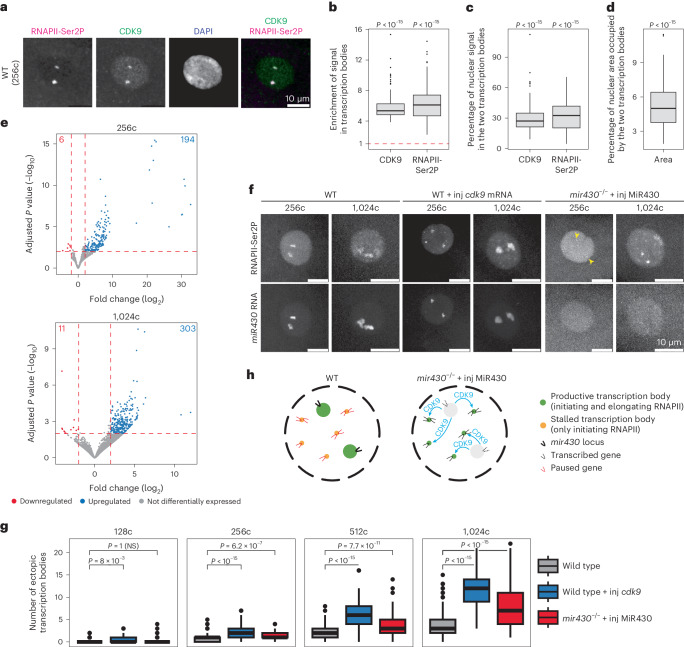
CDK9 sequestration in *mir430* transcription bodies inhibits transcription elongation elsewhere. **a**, Visualization of CDK9 and RNAPII-Ser2P by immunofluorescence in WT nuclei at 256-cell stage. Nuclei are labelled with DAPI. **b**–**d**, Quantification of the enrichment of CDK9 and RNAPII-Ser2P in the two large transcription bodies. The enrichment (observed versus expected signal intensity) (**b**), the percentage of total nuclear signal present in the two large transcription bodies (**c**) and the percentage of the total nuclear area occupied by the two large transcription bodies (**d**) are shown. *N* = 3 biologically independent experiments, *n* = 214 nuclei. Wilcoxon rank-sum test was performed to assess if the log_2_ of the ‘Enrichment of signal in transcription bodies (two-sided)’, the ‘percentage of nuclear signal in two transcription bodies’ (one-sided) and the ‘percentage of nuclear area occupied by two transcription bodies (one-sided)’ are significantly different than zero (*V* = 23,005 for all tests). A test was considered significant when it had a *P* value <0.05. Boxplots show median, quartiles, minimum and maximum, and 1.5× interquartile range. Individual points represent outliers. See Methods for a detailed description of quantification. **e**, Volcano plots showing upregulated and downregulated genes in WT + inj *cdk9* mRNA (*n* = 3 biologically independent samples) versus WT (*n* = 3 biologically independent samples) embryos at the 256-cell stage (top) and the 1,024-cell stage (bottom). Wald test with Benjamini–Hochberg correction was performed, and genes with adjusted *P* values <0.01 were considered significantly differentially expressed. **f**, Visualization of RNAPII-Ser2P with Fabs, and *miR430* RNA with MOVIE in WT, WT + inj *cdk9* mRNA and *mir430*^−/−^ + inj MiR430 embryos across stages. Shown are representative micrographs of individual nuclei at 256-cell and 1,024-cell stages, extracted from a spinning disk confocal microscopy timelapse. See Extended Data Figs. 7–9 for complete cell cycles from 128-cell to 1,024-cell stages. **g**, Quantification of ectopic transcription bodies as shown in **b**. *N* = 3 biologically independent experiments; *n* = 101, 184, 290 and 378 at 128-cell, 256-cell, 512-cell and 1,024-cell stages in WT; *n* = 43, 82, 127 and 179 at 128-cell, 256-cell, 512-cell and 1,024-cell stages in WT + inj *cdk9*; *n* = 45, 65, 97 and 152 at 128-cell, 256-cell, 512-cell and 1,024-cell stages in *mir430*^−/−^ + inj MiR430, respectively. Boxplots show median, quartiles, minimum and maximum, and 1.5× interquartile range. Individual points represent outliers. See Extended Data Fig. 10 and Methods for a detailed description of quantification. **h**, Model for the role of transcription bodies in transcription regulation. In WT nuclei, two large transcription bodies are nucleated by the *mir430* locus. They sequester CDK9 and potentially other factors that are required for pause release, thereby stalling transcription elsewhere in the nucleus in the initiation state. The specific disruption of the two *mir430* transcription bodies leads to a redistribution of CDK9, which results in pause release and the upregulation of genes elsewhere in the nucleus. Source numerical data are available in Source data and on GEO (GSE248237). Source data

**Extended Data Fig. 7:**
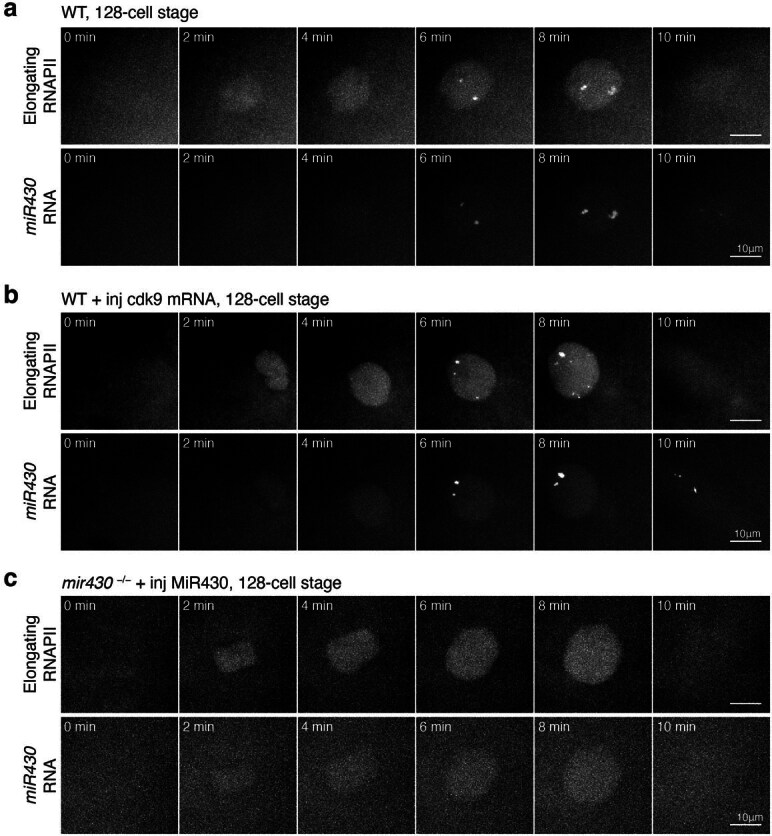
RNAPII-Ser2P signal in WT, WT + inj *cdk9* mRNA and *mir430*^-/-^ + inj MiR430 embryos at 128-cell stage. Visualization of RNAPII Ser2P (with Fabs) and *miR430* RNA (with MOVIE) in WT **(a)**, WT + inj *cdk9* mRNA **(b)**, and *mir430*^-/-^ + inj MiR430 **(c)** embryos at 128-cell stage. Shown are representative images of individual nuclei, extracted from a spinning disk confocal microscopy timelapse. N = 3 biologically independent experiments. Information on quantification can be found in Fig. 6g and Extended Data Fig. 10.

**Extended Data Fig. 8:**
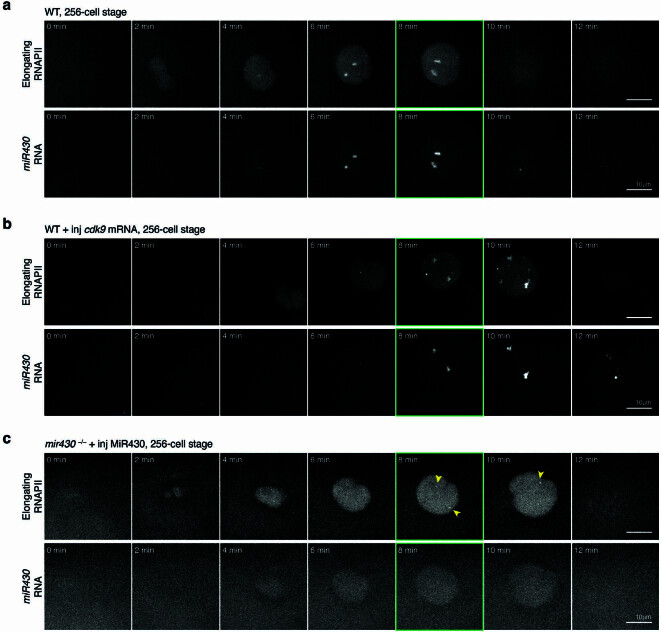
RNAPII-Ser2P signal in WT, WT + inj *cdk9* mRNA and *mir430*^-/-^ + inj MiR430 embryos at 256-cell stage. Visualization of RNAPII Ser2P (with Fabs) and *miR430* RNA (with MOVIE) in WT **(a)**, WT + inj *cdk9* mRNA **(b)**, and *mir430*^-/-^ + inj MiR430 **(c)** embryos at 256-cell stage. Shown are representative images of individual nuclei, extracted from a spinning disk confocal microscopy timelapse. The micrographs shown in the main figure are highlighted in green. N = 3 biologically independent experiments. Information on quantification can be found in Fig. 6g and Extended Data Fig. 10.

**Extended Data Fig. 9:**
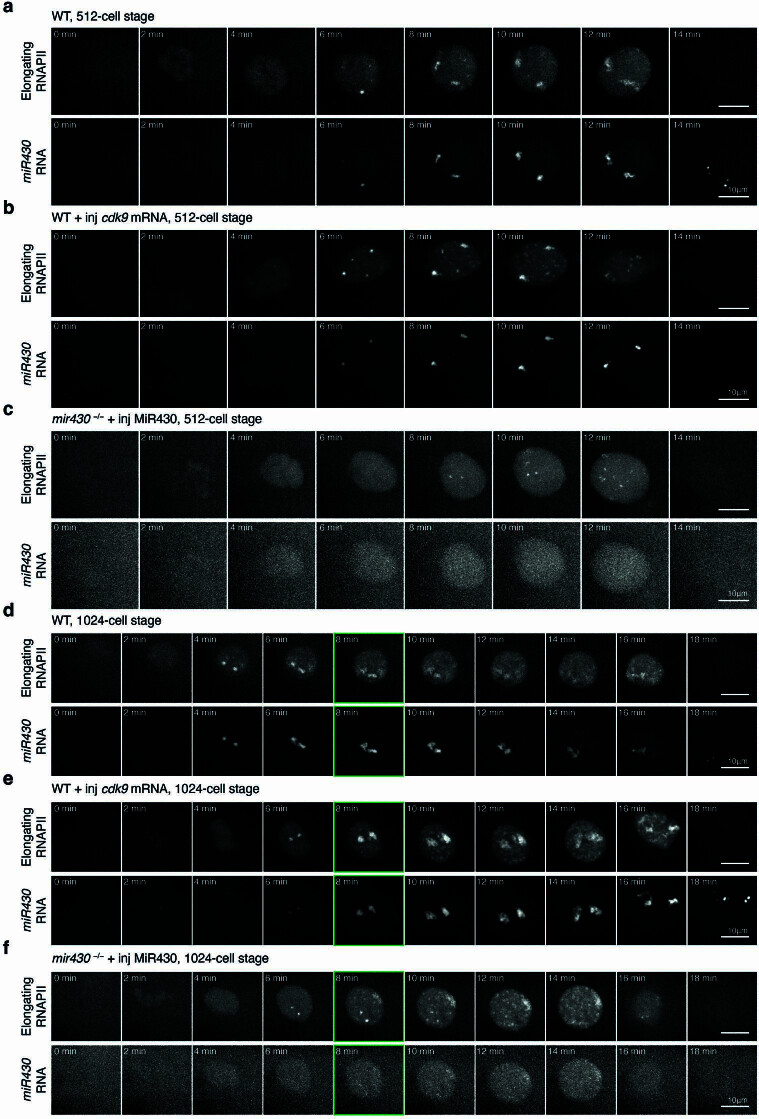
RNAPII-Ser2P signal in WT, WT + inj *cdk9* mRNA and *mir430*^-/-^ + inj MiR430 embryos at 512- and 1024-cell stage. Visualization of RNAPII Ser2P (with Fabs) and *miR430* RNA (with MOVIE) in WT **(a, d)**, WT + inj *cdk9* mRNA **(b, e)**, and *mir430*^-/-^ + inj MiR430 **(c, f)** embryos at 512-cell stage **(a, b, c)** and 1024-cell stage **(d, e, f)**. Shown are representative images of individual nuclei, extracted from a spinning disk confocal microscopy timelapse. N = 3 biologically independent experiments. Information on quantification can be found in Fig. 6g and Extended Data Fig. 10.

**Extended Data Fig. 10:**
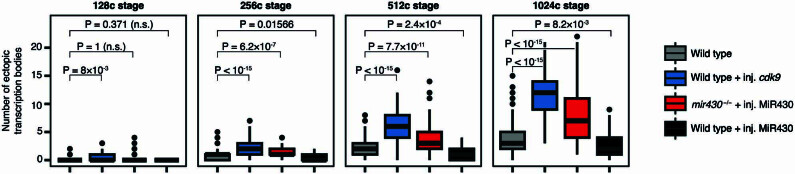
MiR430 injection only modestly affects the number of ectopic transcription bodies. Quantification of ectopic transcription bodies in WT + inj MiR430. Data for WT, WT + inj *cdk9*, and *mir430*^-/-^ + inj MiR430 are the same as in Fig. 6g. N = 3 biologically independent experiments, n = 101, 184, 290 and 378 at 128c, 256c, 512c and 1024c, respectively in WT. n = 43, 82, 127 and 179 at 128c, 256c, 512c and 1024c, respectively in WT + inj *cdk9*. n = 45, 65, 97 and 152 at 128c, 256c, 512c and 1024c, respectively in *mir430*^-/-^ + inj MiR430. n = 14, 33, 75, 92 at 128c, 256c, 512c and 1024c, respectively in WT + inj MiR430. Boxplots show median, quartiles, minimum and maximum, and 1.5x interquartile range. Individual points represent outliers. At each stage, Kruskal-Wallis tests were performed (128c: ***χ***^2^ = 15.685, df = 3, P-value = 0.001315; 256c: ***χ***^2^ = 107.17, df = 3, P-value < 2.2 × 10^−16^; 512c: ***χ***^2^ = 219.57, df = 3, P-value < 2.2 × 10^−16^; 1024c: ***χ***^2^ = 413.81, df = 3, P-value < 2.2 × 10^−16^). When this test was statistically significant (P-value < 0.05), pairwise comparisons with Bonferroni correction were performed using a pairwise Wilcoxon rank-sum test. A comparison was considered significant when adjusted P-value < 0.05, and adjusted P-values were reported using WT as reference. See Methods for a detailed description of quantification. Source numerical data are available in source data. Source data

Our data suggest that transcription bodies sequester a pause release factor. Pause release is regulated by the pTEFb complex, of which CDK9 is the catalytic subunit. We visualized CDK9 in WT embryos by immunofluorescence and found that it is strongly enriched in the two *mir430* transcription bodies (Fig. 6a,b). In fact, CDK9 is almost as enriched in the transcription bodies as its defining component RNAPII-Ser2P. We found that 27.1% of all nuclear CDK9 is in the two transcription bodies (Fig. 6c), while they make up only 5.0% of the total nuclear area (Fig. 6d). If the amount of CDK9 in the nucleus were limiting, and its sequestration in *mir430* transcription bodies would hamper transcription elongation elsewhere in the nucleus, it would be predicted that overexpression of CDK9 in WT embryos would induce the premature expression of ZGA genes and, potentially, the formation of ectopic transcription bodies. To test this, we performed eSLAM-seq to compare gene expression between WT embryos and WT embryos injected with *cdk9* mRNA at the 256- and 1,024-cell stages. In line with our model, we find that overexpressing CDK9 results in the upregulation of many genes (Fig. 6e). Specifically, we find 194 genes upregulated (versus 6 downregulated) at the 256-cell stage, and 303 genes upregulated (versus 11 downregulated) at the 1,024-cell stage. Remarkably, the upregulated genes are, in large part, ZGA genes (83% at 256-cell stage, and 91% at 1,024-cell stage), and there is a good overlap with the genes that are upregulated in the *mir430*^−/−^ + inj MiR430 embryos (53% at the 256-cell stage and 33% at the 1,024-cell stage). Next, we compared the occurrence of ectopic transcription bodies between WT embryos with and without injected *cdk9* mRNA. We visualized RNAPII-Ser2P to detect transcription bodies. To distinguish transcription bodies that are nucleated by the *mir430* locus from ectopic transcription bodies, we also visualized *miR430* transcripts using Morpholino Visualization of Expression (MOVIE^21^). At the 128-cell stage, we detected two prominent *mir430* transcription bodies in nuclei of WT embryos, as well as in WT embryos that were injected with *cdk9* mRNA (Fig. 6f and Extended Data Fig. 7). In the latter, however, we detected additional transcription bodies. Because these lack the *miR430* MOVIE signal and we do not detect them in uninjected WT embryos, we consider these to be ectopic transcription bodies. The ectopic bodies are small and limited in number at the 128- and 256-cell stages, and more prominent at the 512- and 1,024-cell stages (Fig. 6f and Extended Data Figs. 7–9). The abundance of ectopic transcription bodies in WT embryos in which CDK9 is overexpressed phenocopies the abundance of ectopic transcription bodies seen in *mir430*^−/−^ + inj MiR430 embryos. The quantification of ectopic transcription shows that, at all four stages examined, ectopic transcription bodies are significantly more abundant in nuclei in which CDK9 is overexpressed (WT + inj *cdk9* mRNA), or in which the large *mir430* transcription bodies are disrupted (*mir430*^−/−^ + inj MiR430), than in WT embryos (Fig. 6g and Extended Data Fig. 10). Together, this supports our hypothesis that CDK9 is sequestered in *mir430* transcription bodies, thereby limiting CDK9 availability elsewhere in the nucleus.

## Discussion

In this study, we disrupted two large transcription bodies that mark the onset of transcription in zebrafish embryos to study the effect of transcription bodies on gene expression. We have shown that removal of these two large transcription bodies, which are seeded by the *mir430* locus, results in a genome-wide misregulation of gene expression. Surprisingly, the changes in gene expression are not accompanied by any obvious developmental phenotype. While this may be explained, in part, by the quick recovery of gene expression, it is unlikely that the changed temporal dynamics of transcription do not affect development at all. More work will be needed to identify subtle changes in developmental progress, perhaps under challenging conditions. The observation that hundreds of genes are downregulated when two large transcription bodies are disrupted appears to be consistent with a model in which bodies bring together genes^11–13^ and increase the efficiency of transcription^14^. Our experiments, and those of others^30^, however, do not provide any evidence for the consistent colocalization of downregulated genes with the *mir430* bodies and thus argue against a model in which genes consistently colocalize in transcription bodies. We hypothesize that the genes that are downregulated (or their regulatory elements) interact with the *mir430* locus or its associated transcription body more dynamically. To investigate this, genes will need to be labelled live to follow them in nuclear space as they are activated. Our work focussed on the observation that many genes are upregulated upon disruption of the *mir430* transcription bodies. We show that transcription bodies sequester a substantial fraction of the total nuclear amount of the pause release factor CDK9, preventing genes that are not present in the transcription bodies from being transcribed (Fig. 6h). Factor sequestration regulating biological processes has been described before^31,32^ and a recent study showed that the loss of the transcription factor Zelda in *Drosophila* embryos caused RNAPII to relocate from Zelda-containing transcription bodies to the histone locus body^33^. More generally, work in mouse embryos has shown that CDK9 abundance is an important factor in the timing of ZGA^34^. Taken together, our results show that endogenous transcription bodies play an important role in controlling the availability of transcriptional machinery in the nucleus, and consequently transcriptional activity.

## Methods

### Zebrafish and molecular biology approaches

#### Zebrafish husbandry and manipulation

Zebrafish were maintained and raised under standard conditions, and according to Swiss regulations (Canton Vaud, license number VD-H28). WT (TLAB) and mutant (*mir430*^−/−^)^9^ embryos were collected immediately upon fertilization and allowed to develop to the desired stage at 28 °C. *mir430*^−/−^ embryos were rescued by injecting 2 nl of rescue solution (10 μM of MiR430a, MiR430b and MiR430c duplex in 1× siRNA solution (300 mM KCl, 30 mM HEPES, 1 mM MgCl_2_, brought to pH 7.5 with KOH)), as described previously^9,24^. If the chorion was removed chemically, this was done by incubating embryos right after fertilization in 1.5 mg ml^−1^ Pronase E (Sigma-Aldrich, catalogue number 107433) for ~5 min. Developmental stage was determined by morphology (according to ref. ^35^), the distance between nuclei, as well as cell size, cell cycle duration and synchronicity^9^. Fabs were injected into the cell at 0.44 ng per embryo (αRNAPII-Ser2P-Alexa488) or 0.66 ng per embryo (αRNAPII-Ser5P-Cy5). Lissamine-labelled anti-*miR430* morpholino (5′-TCTACCCCAACTTGATAGCACTTTC-3′-Lissamine, Gene Tools) was injected into the cell at 14 fmol per embryo^21^. 4-Thio-UTP (TriLink, tebu-bio GmbH, catalogue number N-1025-1, diluted to 50 mM in 10 mM Tris-HCl pH 7.5) was injected into the yolk at 50 nM per embryo.

#### Genotyping

Embryos (live and fixed) were genotyped using the HotSHOT method^36^.

#### mRNA production and injection

Enhanced green fluorescent protein (eGFP) with three perfect *miR430* target sites in its 3′ UTR (eGFP-3×PT-miR430b) was inserted in the pCS2^+^ plasmid (Addgene) as described previously^24^. The coding sequence of *cdk9* (ENSDART00000065859.8) was amplified from cDNA using SuperScript III (Invitrogen, catalogue number 18080085) and oligodT primers, and inserted in the pCS2^+^ plasmid (Addgene) using restriction-enzyme cloning. mRNA was synthesized using the mMessage mMACHINE Transcription Kit (ThermoFisher, catalogue number AM1340) and purified with the RNeasy MinElute Cleanup Kit (QIAGEN, catalogue number 74204). mRNA was injected into embryos at the one-cell stage in the following amounts: 50 pg of eGFP mRNA and 100 pg of red fluorescent protein (RFP) or *cdk9* mRNA.

#### Preparation of Fabs

Fluorescently labelled Fabs specific to RNAPII-Ser5P and RNAPII-Ser2P were prepared from monoclonal antibodies specific to RNAPII-Ser5P and Ser2P phosphorylation^37–39^. Purified mouse IgG (4 mg) was digested with Ficin (ThermoFisher Scientific) or Papain (ThermoFisher Scientific), and Fabs were purified through protein A-Sepharose columns (Cytiva) to remove Fc and undigested IgG. After passing through desalting columns (PD MiniTrap G-25; Cytiva) to substitute the buffer with PBS, Fabs were concentrated to more than 1 mg ml^−1^ using 10 kDa cutoff filters (Amicon Ultra-0.5 10 kDa; Merck), according to the manufacturer’s instructions.

Fluorescent dye conjugation was conducted using 50 or 100 μg of purified Fab fragments. In a typical reaction, 50 μg of purified Fab was diluted in 45 μl PBS, mixed with 5 μl 1 M NaHCO_3_ (pH 8.3) and then with 0.5 μl of Alexa Fluor 488 (sulfodichlorophenol ester; 10 mg ml^−1^ in DMSO; ThermoFisher Scientific, catalogue number A30052) or 0.5 μl of Cy5 N-hydroxysuccinimide ester (10 mg ml^−1^ in DMSO; cytiva, catalogue number PF11A25001). After incubating for 1 h at room temperature with gentle rotation in the dark, unconjugated fluorescent dye molecules were removed using a PD MiniTrap G-25 column (Cytiva, catalogue number 28918004; pre-equilibrated with PBS). The reaction mixture (50 μl) was applied to a column and 550 μl PBS was applied; the flowthrough fraction was discarded. Dye-labelled Fab fragments were eluted with 500 μl PBS and concentrated to ~1 mg ml^−1^ using a 10-kDa cutoff Amicon Ultracell Centrifuge Filter Unit (Merck, catalogue number UFC5010BK). Fab concentration and Dye:Fab ratio were measured using a Nanodrop (ThermoFisher Scientific). Dye:Fab ratios were between 0.6:1.1 and 1:1. Aliquots of labelled Fabs were bead-loaded into HeLa cells to validate that they distributed as expected and were then stored at 4 °C in the dark.

#### Probe production for in situ hybridization

A digoxigenin (DIG)-labelled antisense probe against *goosecoid* (*gsc*) was generated by in vitro transcription from a linearized plasmid using 10× Transcription buffer (Roche, catalogue number 11465384001), 10× DIG RNA labelling mix (Roche, catalogue number 11277073910), T7 RNA polymerase (Roche, catalogue number 10881775001) and RNase OUT (Invitrogen, catalogue number 10777019). Probe was purified with the RNeasy MinElute Cleanup Kit (QIAGEN, catalogue number 74204), diluted in hybridization buffer and stored at −20 °C.

#### Whole-mount in situ hybridization

Whole-mount in situ hybridization (WM-ISH) was performed as described previously^40^, with some differences. In brief, embryos were fixed at the desired stage in 4% (w/v) paraformaldehyde (PFA) in PBS pH 7.4 overnight at 4 °C. Embryos were then dechorionated manually with sharp forceps (Dumont No. 5) in PBS with 0.1% Tween-20 (PBST) and dehydrated by rinsing them in 50% and then 100% methanol in PBST on a nutator. At this point, embryos could be stored for several weeks at −20 °C. To continue, embryos were rehydrated gradually on a nutator, incubated in hybridization buffer (50% formamide, 1.3× SSC pH 5.0, 5 mM EDTA, 50 μg ml^−1^ torula yeast RNA, 0.2% Tween-20, 0.1% SDS, 100 μg ml^−1^ heparin) for 1 h at 70 °C, and incubated with anti-*goosecoid* DIG-labelled probe overnight at 70 °C. The next day, the probe was washed off with hybridization buffer and embryos were incubated for 15 min in 50% (v/v) hybridization buffer in TBST (25 mM Tris-HCl pH 7.8, 137 mM NaCl, 2.7 mM KCl, 0.1% Tween-20) at 70 °C. After rinsing with TBST, embryos were incubated for 1 h in TBST and then in blocking buffer (2% blocking reagent in maleic acid buffer pH 7.5 (Roche, catalogue number 11096176001) and 10% sheep serum (Sigma-Aldrich, catalogue number S2263) in TBST) at room temperature on a nutator. anti-DIG antibody (Roche, catalogue number 11093274910) was added in a dilution of 1:2,000 and embryos incubated overnight at 4 °C on a nutator. The next day, embryos were washed with TBST and then NTMT (100 mM NaCl, 100 mM Tris-HCl pH 9.5, 50 mM MgCl_2_, 0.1% Tween-20), and stained in NTMT-NBT-BCIP solution (4.5 μl ml^−1^ of NBT (Roche, catalogue number 11383213001) and 3.5 μl ml^−1^ of BCIP (Roche, catalogue number 11383221001) in NTMT) in the dark. Once sufficiently stained, embryos were washed with PBST, and then in 100%, 50% and 30% methanol in PBST at room temperature on a nutator. Embryos were stored at 4 °C in PBST for up to a few days before imaging.

#### Immunofluorescence

Dechorionated embryos were fixed at the desired stage in 4% (w/v) PFA in PBS pH 7.4 overnight at 4 °C. Embryos were then dehydrated by incubating them in 30%, 50% and then 100% methanol in PBS and incubated overnight at −20 °C. To continue, embryos were rehydrated gradually. All following steps were performed while rocking. Embryos were washed several times with PBST (0.8% Triton-X 100 in PBS) and incubated in blocking buffer (10% normal goat serum (NGS) (Sigma-Aldrich, catalogue number G6767) and 4% BSA (Sigma-Aldrich, catalogue number A8022) in PBST) for 4 h at room temperature. Primary antibodies were added in 1% NGS in PBST overnight at 4 °C (anti-CDK9, clone C12F7, Cell Signaling Technology, monoclonal IgG antibody generated in rabbit, 1:200 dilution; anti-RNAPII-Ser2P, clone H5, ab24758, monoclonal IgM antibody generated in mouse, Abcam, 1:1,000 dilution). The next day, embryos were washed with PBST and then incubated with secondary antibodies (anti-rabbit-IgG AlexaFluor488-conjugated, Thermo Scientific, catalogue number A-21206, 1:500 dilution; anti-mouse-IgM AlexaFluor594-conjugated, Thermo Scientific, catalogue number A-21044, 1:500 dilution) in 1% NGS in PBST overnight at 4 °C. Embryos were then washed with PBST, deyolked manually using sharp forceps (Dumont No. 5), and mounted in Vectashield Antifade Mounting Medium with 4,6-diamidino-2-phenylindole (DAPI) (Vector Laboratories, VC-H-1200-10) before imaging them on a Spinning Disk microscope.

### Enriched SLAM-seq

#### Rationale

Early embryos contain large amounts of maternally loaded RNA^19^ that mask nascent RNAs in total RNA sequencing approaches. We therefore developed a method to sensitively detect nascent transcripts. Methods to enrich nascent transcripts have been described previously. Typically, a uracil/UTP analogue (EU/s^4^UTP) is injected at the one-cell stage and is incorporated into newly made RNAs. Such labelled transcripts can then be biotinylated and enriched by pull down^20,41^. SLAM-seq is another approach in which nascent transcripts are labelled by s^4^UTP incorporation, but here the label is used to convert 4-thio-uracils to carboxyamido-methylated 4-thio-uracils, which will be misread as a cytosine during reverse transcription, introducing specific point mutations that enable the identification of reads derived from labelled transcripts by sequencing^42–44^. We combined these two approaches in eSLAM-seq, which is conceptually similar to TT-TimeLapse-Seq and TT-SLAM-Seq^45,46^. Specifically, we used a combination of s^4^UTP labelling^41^ with improved biotinylation and enrichment chemistry^47,48^, followed by SLAM-seq chemistry.

#### Sample preparation

Because of the light-sensitive nature of s^4^UTP, all steps until the treatment of RNA with iodoacetamide (IAA) were performed in the dark. When staging embryos at the stereomicroscope, for example, we used the lowest light setting that allowed us to still see the embryos, and an ultraviolet filter was set between the injection plate and the light source to protect the s^4^UTP. Successive steps were performed protecting the samples from the light by using low light settings in the laboratory room, by working inside a cardboard box and by covering the samples with aluminium foil.

Synchronized s^4^UTP-injected embryos were collected and left to develop to the desired stage. They were collected in as little 0.3× Danieau’s Buffer as possible, homogenized in 500 μl Qiazol (QIAGEN, catalogue number 79306) with a 5 mm stainless steel bead (QIAGEN, catalogue number 69989) using a TissueLyser (30 s at 30 Hz) and stored at −80 °C. Either 100 embryos (256-cell stage) or 50 embryos (1024-cell, Oblong and Sphere stages) were used. Chloroform was added and RNA was extracted by phase separation, using a MaXtract tube (QIAGEN, catalogue number 129046), after which it was precipitated with isopropanol supplemented with 0.2 mM dithiothreitol (DTT) and glycogen. The pellet was washed with 80% ethanol supplemented with 1 mM DTT, air-dried, and resuspended in 40 μl of 1 mM DTT. DNase treatment was performed in 100 μl of 1× DNAse reaction buffer with 4 U of TURBO DNase (ThermoFisher, catalogue number AM2238) for 30 min at 37 °C. The volume was brought to 250 μl with nuclease-free water and RNA was extracted with 25:24:1 phenol-chloroform-isoamyl alcohol (Invitrogen, catalogue number 15593-031) using a MaXtract tube, precipitated with isopropanol supplemented with 0.2 mM DTT, glycogen and sodium acetate. The pellet was washed with 80% ethanol supplemented with 1 mM DTT, air-dried, and resuspended in 40 μl of nuclease-free water.

Labelled RNA was biotinylated in 100 μl of 20 mM HEPES pH 7.4, 1 mM EDTA pH 8.0, 10 ng μl^−1^ MTSEA-biotin-XX (Biotium, catalogue number 90066) and 20% dimethylformamide by incubation at room temperature for 30 min while rotating. The volume was brought to 250 μl with nuclease-free water and RNA was extracted with 24:1 chloroform-isoamyl alcohol (Sigma-Aldrich, catalogue number 227056) using a MaXtract tube, and precipitated with isopropanol supplemented with glycogen and NaCl. The pellet was washed with 80% ethanol, air-dried, and resuspended in 50 μl of nuclease-free water.

To enrich for biotinylated RNA, Dynabeads MyOne Streptavidin C1 beads (Invitrogen, catalogue number 65001) were used. Per sample, 10 μl of beads were washed twice in nuclease-free water and twice in high-salt wash buffer (100 mM Tris-HCl pH 7.4, 10 mM EDTA pH 8.0, 1 M NaCl, 0.05% Tween-20), incubated for at least 1 h at room temperature in bead-blocking buffer (40 ng μl^−1^ glycogen in high-salt wash buffer) and washed twice with high-salt wash buffer. RNA was denatured at 65 °C for 10 min, incubated for 5 min on ice and then added to the blocked beads. After incubation for 15 min while rotating at 30 r.p.m., the supernatant was removed and beads were washed three times with high-salt wash buffer, twice with 5.9 M guanidinium chloride (with 5-min incubation at room temperature between consecutive washes), once with TE buffer and three times with TE buffer (with 5-min incubation at 55 °C between consecutive washes). Bead-bound RNA was finally released by adding 25 μl of freshly made elution buffer (100 mM DTT, 20 mM HEPES pH 7.4, 1 mM EDTA pH 8.0, 100 mM NaCl, 0.05% Tween-20) twice. The volume was brought to 100 μl with nuclease-free water and RNA was precipitated with isopropanol supplemented with 0.2 mM DTT, glycogen and NaCl. The pellet was washed with 80% ethanol supplemented with 1 mM DTT, air-dried and resuspended in 20 μl of 1 mM DTT.

IAA-mediated alkylation was performed in 10 mM IAA (freshly dissolved in 100% ethanol, BioUltra, catalogue number I1149), 50 mM NaPO_4_ pH 8.0 and 50% dimethylsulfoxide (DMSO) for 15 min at 50 °C. Samples were then transferred to ice and the reaction stopped by quickly adding 1.4 μl of 1 M DTT. RNA was precipitated with 100% ethanol supplemented with glycogen and sodium acetate overnight. The pellet was washed with 80% ethanol, air-dried and resuspended in 8.5 μl of nuclease-free water.

Libraries were prepared with the TruSeq stranded mRNA Kit (Illumina, catalogue number 20020594) and TruSeq RNA UD Indexes (Illumina, catalogue number 20022371). The polyA enrichment step was not performed. Fragmentation was performed for 1 min at 94 °C. Paired-end 150 bp sequencing was performed on an Illumina NovaSeq 6000 System, loading each library on two lanes (Illumina Reagent Kit v.1.5). Sequencing data were demultiplexed with bcl2fastq v.2.20.

#### Quality controls

To assess the integrity of total RNA (Extended Data Fig. 3c, left panel), 1 μg per sample was diluted in 2× RNA Gel Loading Dye (Thermo Scientific, catalogue number R0641), denatured for 90 s at 85 °C, chilled for 5 min on ice and loaded onto an agarose gel alongside RiboRuler High Range RNA Ladder (ThermoFisher, catalogue number SM1823). The presence of two prominent bands (rRNA) are an indicator of RNA integrity.

To test the specificity of s^4^UTP biotinylation (Extended Data Fig. 3c, right panel), the RNA was then transferred from the gel onto a Nylon Hybond-N+ membrane (Amersham, catalogue number RPN203B) by capillary transfer overnight using a Whatman Turboblotter in 20× SSC according to the manufacturer’s instructions. After drying, the membrane was crosslinked twice with 120 mJ cm^−2^ using a ultraviolet Stratalinker 1800 (Stratagene). The membrane was then incubated for 20 min in Blocking Buffer (10% SDS and 1 mM EDTA in 1× PBS pH 7.5), for 1 h in 1:10,000 Streptavidin-HRP conjugate (Perkin, catalogue number NEL750) in Blocking buffer, two times for 10 min in 10% SDS in 1× PBS pH 7.5, two times for 10 min in 1% SDS in 1× PBS pH 7.5, and two times for 10 min in 0.1% SDS in 1× PBS pH 7.5, all while nutating^49^. Finally, the signal was visualized with enhanced chemiluminescence western blotting detection reagents (Amersham, catalogue number RPN2106) according to the manufacturer’s instructions, and documented with a Fusion FX (Vilber).

### Bioinformatic analyses

#### eSLAM-seq

To map the raw sequencing data, we concatenated Fastq files that came from the same library but that were run on different lanes with the ‘cat’ command (GNU core utilities, linux terminal), trimmed them using Trimmomatic (Paired-end mode, LEADING:3 TRAILING:3 SLIDINGWINDOW:4:15 MINLEN:36)^50^ and mapped them using HISAT-3N (–base-change T,C–repeat–rna-strandness RF–fr–no-discordant)^51^, which is an HISAT variant designed specifically for methods like SLAM-Seq that introduce specific point mutations. Uniquely mapped fragments were filtered with SAMtools^52^ and mate information was fixed with Picard FixMateInformation (–IGNORE_MISSING_MATES FALSE)^53^.

To distinguish between naturally occurring single nucleotide polymorphisms (SNPs) and SLAM-seq-specific mismatches, we first identified naturally occurring SNPs by analysing four IAA-minus samples (one per stage) in which the IAA treatment—and therefore the base conversion—was not performed. The bam files were converted to a suitable format with the SAMtools mpileup function and then SNPs were identified by Varscan pileup2snp (–min-coverage 20–min-reads2 5–min-avg-qual 15–min-var-freq 0.25–*P* value 0.01). The union of the SNPs that were identified was used as a consensus list. Then the splbam.py script from pulseR^54^ was used to select fragments with at least one SLAM-seq-specific mismatch.

To assess gene expression levels of annotated genes, raw read counts were obtained using FeatureCounts (-p–countReadPairs -B–minOverlap 10 -s 2 -t exon -g gene_id)^55^. Normalization, principal component analysis (PCA) and call for differential expression (alpha = 0.01 and log_2_(fold change) threshold = 2) was performed using DESeq2 (ref. ^56^). When performing PCA, only the top 500 genes (selected by highest variance in expression levels) were considered. When counting the number of differentially expressed genes, mitochondrial and *mir430*-coding genes were excluded.

#### Motif analysis

To assess the presence and enrichment of specific motifs in promoters of upregulated genes, we started with the non-redundant JASPAR 2022 Vertebrate core database. From this, only motifs whose cognate transcription factors are expressed during ZGA (Riboprofiling dataset, >10 rpkm^27^) were kept. The Nanog motif^26^ and Pou5f1 motif^28^ were added, because it is known that these factors are important during zebrafish ZGA. Next, we extracted promoter sequences (4,000 bp centred around the TSS for upregulated genes as well as non-expressed genes (fewer than ten raw reads in total across all samples, background group). Motif enrichment was then assessed using SEA (MEME Suite^57^). To estimate depletion of motifs, the background and input sample were inverted. Only motifs with a *P* value < 0.05 and an E-value ≤ 10 were considered.

#### Promoter accessibility analysis

To analyse promoter accessibility of the upregulated genes in WT, we used an ATAC-seq dataset that we had generated previously^26^. Replicates were merged and the signal in 4,000 bp centred around the TSS was plotted using Deep Tools^58^. Promoter signal was used to rank all promoters by accessibility and to assess their enrichment amongst the most accessible promoters. The 716 most expressed (mean transcripts per million) genes, non-expressed genes and non-differentially expressed genes (excluding mitochondrial genes and *mir430*-coding genes) were used as a comparison.

#### Gene enrichment analysis

The genomic distribution of gene sets was visualized using karyograms, and their chromosome-enrichment was calculated by dividing the fraction of upregulated or downregulated genes on a chromosome by the fraction of all genes on that chromosome. The significance of the enrichment was determined using the hypergeometric distribution, and *P* values were corrected for multiple testing with the Benjamini–Hochberg method.

### Graph plotting

Graphs were generated using R (v.4.0.5)^59^ with the ggplot2 (ref. ^60^) and Ggbio package^61^. In dotplots, the red rhombuses represent the median. In boxplots, the centre line represents the median, the box limits represent the upper and lower quartiles, the whiskers represent the 1.5× interquartile range and individual points represent outliers.

### DNA-fluorescence in situ hybridization

#### Gene selection

To investigate whether downregulated genes consistently localize to the *mir430* transcription bodies, we investigated the nuclear distribution of ten downregulated genes (*otud7b*, *nipblb*, *ncoa2*, *tln2a*, *rai14*, *iars1*, *hpca*, *psmd1*, *cbl*, *nol4la*) with respect to the *mir430* locus, as well as the position of ten upregulated genes (*epha2b*, *flncb*, *nr5a2*, *dhx15*, *pcdh1b*, *waplb*, *ttn.2*, *cdc14b*, *rasa1a*, *plcg1*) as a control. Genes were selected based on expression level, genomic location and number of probes covering a gene. Regarding the expression change in the eSLAM-seq dataset, only genes with an absolute log_2_ fold change greater than 2.0 (*mir430*^−/−^ + inj MiR430 versus WT) were considered.

#### Probe design for upregulated and downregulated genes

A genome-scale DNA-FISH probe collection for zebrafish was downloaded from the PaintSHOP^62^ (https://github.com/beliveau-lab/PaintSHOP_resources; danRer11 newBalance). From this collection, only unique sequences complementary to the selected upregulated or downregulated genes were selected for Oligopaint library design. We required genes to be at least 40 kb in length, and to be covered by at least 400 probes. In a few cases of very long genes, only the 5′ regions, including the promoters, were selected for labelling. We selected for the probe density to be 10 ± 1 probe per kilobase for each gene. To avoid potential colocalization because of proximity on the same chromosome, we selected the genes of a single group (up or down) to be located on different chromosomes (except for two upregulated genes (*plcg1* and *epha2b*) that are both located on chr23 but are separated by a genomic distance of ~22 Mb). Within the library, each oligonucleotide is a concatenation of six sequences (in the 5′–3′ direction): forward priming sequence, reverse transctiptase priming sequence, gene-specific readout sequence, genomic target sequence, group-specific readout sequence and reverse priming sequence (Fig. 3c,d). Taken together, each oligonucleotide within the Oligopaint library is 130–137 nt long. The priming sequences, described previously^63^, are common for all oligonucleotides. The gene-specific and group-specific readout sequences, also described previously^63^, are common for each oligo corresponding to the same single gene, or to the same group of genes (upregulated or downregulated), respectively. The genomic target sequence comes from the PaintSHOP collection and is unique for each oligonucleotide. The library itself, consisting of 12,472 oligonucleotides, was ordered from GenScript as an ‘Oligo Pool’. The priming and readout sequences and the complete Oligopaint library are provided in Supplementary Table 2.

#### Probe design for the *mir430* locus

Because of the repetitive nature of the *mir430* locus, a single DNA-FISH probe is sufficient to visualize *mir430* DNA. The genomic target sequence complementary to *mir430* was generated using PaintSHOP software using default parameters and with genomic coordinates of the *mir430* locus (chr4:28675373–28710158; danRer11 genome assembly) as input. In the probe, this sequence is flanked by priming sequences, which are the same as in the Oligopaint library for misregulated genes (‘Probe design for upregulated and downregulated genes’). As *mir430* is the only target of this probe, the readout sequences are not necessary. The full sequence of the *mir430* probe is provided in Supplementary Table 2.

#### Probe generation

DNA-FISH probes were produced as described previously^64^. Briefly, stocks of the Oligopaint library for the misregulated genes, as well as the *mir430* probe, were diluted 1:50 in nuclease-free water; 5 μl of these dilutions were used as templates in quantitative PCR (qPCR) reactions using Phusion Hot Start Flex 2× Master Mix (New England Biolabs, catalogue number M0536S). The probes for *mir430*, upregulated and downregulated genes were amplified in three separate reactions using different reverse primers complementary either to the group-specific readout sequences (in the case of the upregulated and downregulated genes) or to the reverse priming sequence (in the case of *mir430*), in each case with a tail encoding the T7 promoter sequence (Fig. 3c,d). qPCR products were column-purified using the DNA Clean & Concentrator-5 kit (Zymo Research, catalogue number D4014) and the purified qPCR products were used for in vitro transcription using HiScribe T7 Quick High Yield RNA Synthesis Kit (New England Biolabs, catalogue number E2050S). The total products of the in vitro transcription reaction were used as templates for the reverse transcription reactions using the Maxima H Minus Reverse Transcriptase kit (ThermoFisher Scientific, catalogue number EP0753). The primer used for the reverse transcription always has the same sequence but is fused to different fluorophores (6-FAM, Cy3, Cy5), so that the three final probe sets can be visualized simultaneously. Following reverse transcription, the RNA remaining in the products was digested by the addition of 50 μl of a 1:1 mix of 0.5 M EDTA and 1 M NaOH and incubation at 95 °C for 10 min. The products were then column-purified using the DNA Clean & Concentrator-25 kit and eluted in 30 μl of nuclease-free water each. The probes were stored at 4 °C protected from light. The qPCR and reverse transcriptase primers used for probe generation are provided in Supplementary Table 2.

#### DNA-FISH procedure

DNA-FISH experiments were performed as described previously^65^, with some adaptations to zebrafish embryos. Briefly, dechorionated embryos were fixed at the 256-cell stage in 4% (v/v) PFA (Electron Microscopy Sciences, catalogue number 15710) in 1× PBS (Gibco, catalogue number 10010-015) overnight at 4 °C, while rotating. The embryos were deyolked mechanically by shaking at 1,400 r.p.m. in PBST (0.2% Tween-20 in 1× PBS) at room temperature for 30 min. The deyolked embryos were then washed three times for 5 min with PBST and permeabilized for 30 min in permeabilization solution (0.5% Triton-X 100 in 1× PBS) at room temperature. The embryos were then washed for 2 min in PBST and incubated in 0.1 N HCl for 5 min. The embryos were then washed three times (1 min, 2 min, 2 min) in 2× SSCT (0.2% Tween-20 in 2× SSC (3 M NaCl, 0.3 M sodium citrate, pH 7.0)) and incubated in the prehybridization solution (50% formamide (deionized) in 2× SSCT) for 4 days at room temperature. The embryos were then incubated again in the prehybridization solution for 2 h at 60 °C. Following prehybridization, the embryos were denatured in 100 μl hybridization solution (2× SSC, 50% formamide, 10% dextran sulfate) containing 5 μl of each of the three fluorescent probes (*mir430*, upregulated and downregulated genes) at 90 °C for 5 min, followed by an overnight hybridization at 37 °C with shaking (1,100 r.p.m.), protected from light. Following hybridization, the embryos were incubated in 2× SSCT at 60 °C for 15 min, then in 2× SSCT at room temperature for 10 min and finally in 0.2× SSC at room temperature for 10 min. All washes were performed in the dark. Following the washes, the embryos were mounted in Vectashield Antifade Mounting Medium with DAPI (Vector Laboratories, catalogue number VC-H-1200-10) before imaging them on a spinning disk microscope.

#### DNA-FISH analysis

Using a custom-written Fiji script (available at https://github.com/CoulonLab/FISHingRod), nuclei were segmented using the DAPI channel. The segmentation was curated manually (removal of debris, separation of several nuclei segmented as one object) to ensure that only interphase nuclei were retained for further analyses. Then, using custom-written Python scripts (available at https://github.com/CoulonLab/FISHingRod), DNA-FISH spots were detected within segmented nuclei. This happens by applying band-pass filtering on the raw images, which is followed by detection of local three-dimensional (3D) maxima and subsequently an iterative 3D Gaussian mask fit^66^. This approach yields fluorescence intensities and 3D coordinates of the spots. Since ten genes of each group were visualized in diploid nuclei, the 20 brightest of the detected spots within each channel (green for upregulated genes, red for downregulated genes) were selected for the 3D distance analysis. For *mir430*, the two brightest spots were selected.

### Microscopy

#### Fluorescence microscopy

To determine MiR430 activity using the GFP sensor assay, injected embryos were dechorionated manually at 24 h postfertilization (hpf) and kept in Petri dishes. Fluorescence signal was measured on an OLYMPUS SZX16 stereomicroscope with a MicroPublisher v.5.0 RTV-R-CLR-10C colour camera (×5 magnification, 400 ms exposure time).

#### Brightfield imaging

To assess developmental progression until 24 hpf, a mould^67^ was used to create agarose wells using as little as possible 1% agarose in 0.3× Danieau’s solution in an Ibidi μ-Dish 35 mm high (catalogue number 81158). The dish was mounted on a 28 °C heated stage (by a Warner automatic temperature controller) and dechorionated embryos were loaded to the single wells at the two-cell stage. Videos with 10-min time intervals were taken on a Zeiss Axio Observer.Z1 microscope (Zeiss ×5/0.25 Fluar air objective, Zeiss AxioCam MRm Monochrome CCD camera). A z-stack (400 μm in nine steps) was taken for each embryo. A lid was used to reduce evaporation of buffer during imaging.

To assess developmental progression of live embryos past somitogenesis, snapshots of manually dechorionated embryos were taken at 24 hpf, 48 hpf and 72 hpf on a Zeiss Axio Observer.Z1 microscope (Zeiss ×2.5/0.08 EC Plan-Neofluar air objective, Zeiss AxioCam ICc 1).

The gene expression pattern in fixed embryos after WM-ISH was visualized on a LEICA M165C stereomicroscope with LEICA MC170 HD colour camera (×4 magnification). Embryos were oriented such that the dorsal region of the embryo was visible.

To determine the cell cycle length, a mould^67^ was used to create agarose wells using 1% agarose in 0.3× Danieau’s solution in a Petri dish. The dish was mounted on a 28 °C heated stage (Okolab LEICA10447342-ROUND-GLASS, controlled by a Okolab H401-T-PENNY temperature controller) and dechorionated embryos were loaded to the single wells at the eight-cell stage and moved gently to a lateral position with tweezers. Videos with 30-s time intervals were taken on a Leica M205 FCA stereomicroscope (×0.78 magnification, Leica DFC7000T camera).

#### Spinning disk microscopy—live

For imaging on a spinning disk microscope, dechorionated embryos were mounted at 32-cell stage in small droplets of 1% low-melting agarose (Invitrogen, catalogue number 16520-050) in 75% v/v 0.3× Danieau’s solution and 25% v/v Optiprep Density Gradient Medium (Sigma-Aldrich, catalogue number D1556)^68^ on a Ibidi μ-Dish 35 mm high (catalogue number 81158). Embryos were brought closer to the coverslip surface by keeping the dish upside down until the agarose solidified (~20 min). More agarose was then added to avoid drying out. Transcription bodies in live embryos were imaged on a Nikon eclipse Ti2 with a Yokogava CSU-W1 Spinning Disk unit at 28 °C (Okolab temperature control and microscope enclosure). Multiposition, *z*-stack (20 μm in 41 steps, NIDAQ Piezo Z) timelapse (2 min time interval) imaging was performed with a Nikon ×60/1.2 Plan Apochromat VC water objective. Images were captured in parallel on two Photometrics Prime 95B cameras with 100 ms exposure time at 10% laser intensity.

#### Spinning disk microscopy—fixed

Immunofluorescence signal (CDK9, RNAPII-Ser2P and DAPI) was imaged on a Nikon eclipse Ti2 with a Yokogava CSU-W1 Spinning Disk unit. Z-stacks (20 μm in 41 steps, NIDAQ Piezo Z) were taken with a Nikon ×60/1.2 Plan Apochromat VC water objective. Each channel was captured in series on a Photometrics Prime 95B camera with 100 ms exposure time at 10% laser intensity each.

The DNA-FISH samples were imaged on a Nikon eclipse Ti2 microscope with a Yokogawa CSU-W1 spinning disk unit. Images were taken with a Nikon ×100/1.35 CFI SR HP Apochromat Lambda S silicone oil objective. For each biological replicate, two to three fields of view were acquired, with each field of view being a *z*-stack spanning the volume of a whole embryo with steps of 0.3 μm. Each channel was captured on a Photometrics Prime 95B camera with 100 ms exposure time at 10% laser intensity for DAPI and 300 ms exposure time at 100% laser intensity for FAM, Cy3 and Cy5.

### Image processing and analysis

#### Software used for image analysis

Microscopy image handling was done using FIJI^69^. Further data processing was carried out using R v.4.0.5 (ref. ^59^) and plots were generated with the ggplot2 package^60^.

#### Analysis of eGFP reporter and RFP control

To assess *mir430* activity, mean fluorescence of eGFP (targeted by MiR430) and RFP (not targeted, and used as a normalizer) were measured in a rectangle of equal size in the head region and a background region outside of the embryo. The signal ratio was then calculated as follows: (eGFP_head_ − eGFP_background_)/(RFP_head_ − RFP_background_).

#### Detection and classification of *mir430* and ectopic transcription bodies

We identified transcription bodies by the presence of RNAPII-Ser2P signal. To classify them into *mir430* and ectopic transcription bodies, we analysed *miR430* MOVIE signal. In case of overlap between RNAPII-Ser2P and MOVIE signal, bodies were classified as *mir430* transcription bodies. In case of no overlap between RNAPII-Ser2P and MOVIE signal, bodies were classified as ectopic transcription bodies. We used FIJI plugin ComDet^70^, which detects spots using consistent intensity thresholding parameters that were set manually and used for all experiments (Supplementary Data Fig. 1a). Please note that, due to the size and amorphous shape of the *mir430*-nucleated transcription bodies, ComDet can detect these bodies as several spots. With the aid of the MOVIE channel, we still count them as single bodies (Supplementary Data Fig. 1b). Furthermore, the two *mir430*-nucleated transcription bodies can fuse to generate one large transcription body. If at the beginning of interphase two bodies could be observed that later fused (in other words, if the fusion event can be observed), the cell will still be classified as having two *mir430*-nucleated transcription bodies (Supplementary Data Fig. 1c). Finally, we note that frames taken after the disassembly of the nuclear envelope were not analysed because, at that time, transcription bodies also disassembled (Supplementary Data Fig. 1d).

#### Detection and classification of transcription bodies by transcription state

Nuclei were segmented using the RNAPII-Ser5P channel, and RNAPII-Ser5P and Ser2P bodies detected using ComDet. This was used to classify transcription bodies into initiating, initiating-elongating and elongating bodies. We then identified and removed *mir430* transcription bodies from the analysis based on their morphological features (early appearance during cell cycle, larger size, growth behaviour) using the RNAPII-Ser2P channel. Here too, frames taken after the disassembly of the nuclear envelope were not analysed. For each nucleus, *t* = 0 is set as the first frame in which spots appear, and the last timepoint is set as the last frame before prophase. Nuclei in which spots were detected for only one frame were discarded.

#### Quantification of CDK9 and RNAPII-Ser2P enrichment in transcription bodies by immunofluorescence

To assess the enrichment of CDK9 in the two *mir430*-nucleated transcription bodies, nuclei that showed both CDK9 and RNAPII-Ser2P signal in the two transcription bodies were selected. Nuclei and transcription bodies were segmented manually using the DAPI and RNAPII-Ser2P channel, respectively, and the area and mean intensity was measured inside of these masks. We then calculated, for each nucleus, the fraction of area occupied by the two bodies as follows:

$${\rm{PercentageArea}}=100\times \frac{{{\rm{Area}}}_{{\rm{Body1}}}+{{\rm{Area}}}_{{\rm{Body2}}}}{{{\rm{Area}}}_{{\rm{Nucleus}}}}$$

To correct for background staining, the mean intensity in no-primary control nuclei was measured as well, and the average mean intensity of all analysed nuclei was determined.

For both the CDK9 and RNAPII signal, we calculated, for each nucleus, the fraction of signal present in the two bodies as follows:

$$\begin{array}{l}{\rm{PercentageIntensity}}\\=100\times \displaystyle\frac{{{\rm{Area}}}_{{\rm{Body1}}}\times {{\rm{MeanIntensity}}}_{{\rm{Body}}1}+{{\rm{Area}}}_{{\rm{Body}}2}\times {{\rm{MeanIntensity}}}_{{\rm{Body}}2}}{{{\rm{Area}}}_{{\rm{Nucleus}}}\times [{{\rm{MeanIntensity}}}_{{\rm{Nucleus}}}-{{\rm{MeanIntensity}}}_{{\rm{Nucleus}},{\rm{NoPrimary}}}]}\end{array}$$

For both the CDK9 and RNAPII signal, we calculated, for each nucleus, the observed over expected mean intensity in the two bodies as follows:

$$\begin{array}{l}{\rm{IntensityEnrichment}}=\\\displaystyle\frac{\begin{array}{c}[{{\rm{Area}}}_{{\rm{Body1}}}\times {{\rm{MeanIntensity}}}_{{\rm{Body}}1}+{{\rm{Area}}}_{{\rm{Body}}2}\times {{\rm{MeanIntensity}}}_{{\rm{Body}}2}]\\/[{{\rm{Area}}}_{{\rm{Body1}}}+{{\rm{Area}}}_{{\rm{Body2}}}]\end{array}}{{{\rm{MeanIntensity}}}_{{\rm{Nucleus}}}-{{\rm{MeanIntensity}}}_{{\rm{Nucleus}},{\rm{NoPrimary}}}}\end{array}$$

### Sample size and statistics

A minimum of three biological replicates (*N*) was acquired for each experimental condition. Each biological experiment was obtained from different and independent embryo clutches. Lowercase *n* refers to the number of embryos or nuclei for each experimental condition. No statistical methods were used to predetermine sample sizes. Rather, sample size was determined based on similar studies in the field.

To determine whether there is a statistically significant difference between the three genotypes in the degree of MiR430 activity as determined by the eGFP-Sensor assay (Fig. 1h), the time of completion of Epiboly (Fig. 1j) and the time of appearance of Kupffer’s vesicle (Fig. 1k), Kruskal–Wallis tests were performed. If this test was statistically significant (*P* value < 0.05), pairwise comparisons with Bonferroni correction were performed using a pairwise Wilcoxon rank-sum test. A comparison was considered significant when adjusted *P* value < 0.05. Dotplots were used to display data, and adjusted *P* values were reported using WT as reference.

To determine whether there is a statistically significant difference in cell cycle length between WT, *mir430*^−/−^ and *mir430*^−/−^ + inj MiR430 (Extended Data Fig. 2b), Kruskal–Wallis tests were performed at each stage, none of which was significant (*P* value < 0.05).

To determine whether the log_2_ of the ‘enrichment of CDK9 signal in transcription bodies’ (Fig. 6b), the ‘percentage of nuclear CDK9 signal in two transcription bodies’ (Fig. 6c) and the ‘percentage of nuclear area occupied by two transcription bodies’ (Fig. 6d) are significantly different from zero, a Wilcoxon rank-sum test was performed. A test was considered significant when the *P* value < 0.05.

To determine whether there is a statistically significant difference in the number of detected ectopic transcription bodies between WT, *mir430*^−/−^ + inj MiR430, WT + inj *cdk9* mRNA and WT + inj MiR430 (Fig. 6g and Extended Data Fig. 10), a pairwise Wilcoxon rank-sum test with Bonferroni correction was performed at each stage. A comparison was considered significant when adjusted *P* value < 0.05.

### Inclusion and ethics statement

We support inclusive, diverse and equitable conduct of research.

### Reporting summary

Further information on research design is available in the Nature Portfolio Reporting Summary linked to this article.

## Online content

Any methods, additional references, Nature Portfolio reporting summaries, source data, extended data, supplementary information, acknowledgements, peer review information; details of author contributions and competing interests; and statements of data and code availability are available at 10.1038/s41556-024-01389-9.

### Supplementary information

Supplementary Information Supplementary Fig. 1.

Reporting Summary

Supplementary Table 1 List of differentially expressed genes in *mir430*^−/− ^+ inj MiR430 versus WT at all four stages and list of differentially expressed genes in WT + inj MiR430 versus WT as well as WT + inj *cdk9* mRNA versus WT, at 256c and 1024c.

Supplementary Table 2 Priming sequences, readout sequences, primers for qPCR and reverse transcription (DNA-FISH) and complete list of oligopaint library sequences (DNA-FISH).

### Source data

Source Data Figs. 1 and 4–6 and Extended Data Figs. 2 and 10 Numerical source data.

Source Data Extended Data Fig. 3 Unprocessed images.

## Data Availability

Sequencing data have been uploaded to Gene Expression Omnibus (GEO) (GSE248237). Imaging data are available upon request. All other data are available in the main text or the Extended Data. Published data have been retrieved from GEO (GSE130944) and JASPAR database (2022 vertebrate core database, https://jaspar.elixir.no). Source data are provided with this paper.

## Code availability

Scripts for the analysis of sequencing and imaging data are available on GitHub (https://github.com/martino-ugolini (ref. ^71^)).
