# Supplementary material for: Transcription bodies regulate gene expression by sequestering CDK9
Source: Nat Cell Biol. 2024 Apr 8;26(4):604–12. doi: 10.1038/s41556-024-01389-9 (PMC11021188; doi:10.1038/s41556-024-01389-9)
Supplement: Supplementary file 1 — Supplementary Fig. 1. [file 41556_2024_1389_MOESM1_ESM.pdf]

nature cell biology

Article https://doi.org/10.1038/s41556-024-01389-9

Transcription bodies regulate gene expression by sequestering CDK9

In the format provided by the authors and unedited

Supplementary Figure 1

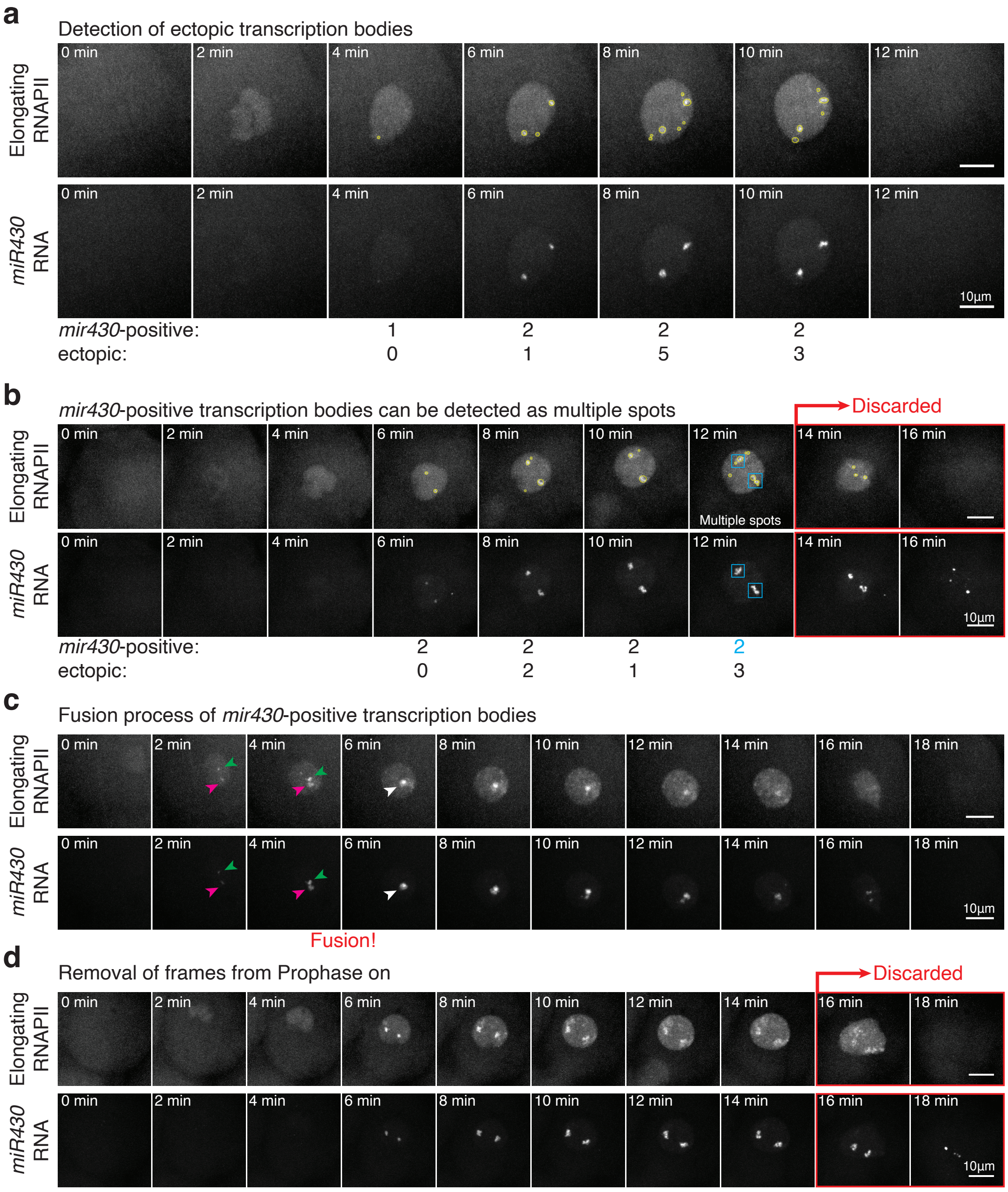

Supplementary Figure 1. Classification of *mir430* and ectopic transcription bodies. a. Transcription bodies are identified by detecting elongating RNAPII signal. If transcription bodies overlap with MOVIE signal (detecting *miR430* RNA) they are classified as *mir430*, and if not, they are classified as ectopic transcription bodies. **b.** Occasionally *mir430* transcription bodies don't have a round shape and are therefore detected as multiple spots. With the aid of the MOVIE channel, we still count them as single bodies (blue rectangles). **c.** When *mir430*

transcription bodies fuse during the cell cycle, the nucleus will still be considered to have generated two transcription bodies. **d.** During the disassembly of the nuclear envelope transcription stops and transcription bodies tend to dissolve, making their quantification impossible. Stages after prophase are therefore discarded.
