# Supplementary material for: Transcription bodies regulate gene expression by sequestering CDK9
Source: Nat Cell Biol. 2024 Apr 8;26(4):604–12. doi: 10.1038/s41556-024-01389-9 (PMC11021188; doi:10.1038/s41556-024-01389-9)
Supplement: Supplementary file 2 — Reporting Summary [file 41556_2024_1389_MOESM2_ESM.pdf]

# nature portfolio

Corresponding author(s): Prof. Nadine L. Vastenhouw

Last updated by author(s): Feb 21, 2024



# Reporting Summary

Nature Portfolio wishes to improve the reproducibility of the work that we publish. This form provides structure for consistency and transparency in reporting. For further information on Nature Portfolio policies, see our Editorial Policies and the Editorial Policy Checklist.

## Statistics

For all statistical analyses, confirm that the following items are present in the figure legend, table legend, main text, or Methods section.

| n/a | Confirmed | |
|---|---|---|
| ☐ | ☒ | The exact sample size (*n*) for each experimental group/condition, given as a discrete number and unit of measurement |
| ☐ | ☒ | A statement on whether measurements were taken from distinct samples or whether the same sample was measured repeatedly |
| ☐ | ☒ | The statistical test(s) used AND whether they are one- or two-sided<br>*Only common tests should be described solely by name; describe more complex techniques in the Methods section.* |
| ☒ | ☐ | A description of all covariates tested |
| ☐ | ☒ | A description of any assumptions or corrections, such as tests of normality and adjustment for multiple comparisons |
| ☐ | ☒ | A full description of the statistical parameters including central tendency (e.g. means) or other basic estimates (e.g. regression coefficient) AND variation (e.g. standard deviation) or associated estimates of uncertainty (e.g. confidence intervals) |
| ☐ | ☒ | For null hypothesis testing, the test statistic (e.g. *F*, *t*, *r*) with confidence intervals, effect sizes, degrees of freedom and *P* value noted<br>*Give P values as exact values whenever suitable.* |
| ☒ | ☐ | For Bayesian analysis, information on the choice of priors and Markov chain Monte Carlo settings |
| ☒ | ☐ | For hierarchical and complex designs, identification of the appropriate level for tests and full reporting of outcomes |
| ☒ | ☐ | Estimates of effect sizes (e.g. Cohen's *d*, Pearson's *r*), indicating how they were calculated |

*Our web collection on statistics for biologists contains articles on many of the points above.*

## Software and code

Policy information about availability of computer code

| | |
|---|---|
| Data collection | No software was used to collect data in this study. |
| Data analysis | Microscopy data were analyzed using FIJI (1.53q) and R (4.3.1), while sequencing data were analyzed using a multitude of published and well-established softwares. The working steps are described in detail in the Methods section of the manuscript, and the scripts are available on GitHub (@martino-ugolini and https://github.com/CoulonLab/FISHingRod). |

For manuscripts utilizing custom algorithms or software that are central to the research but not yet described in published literature, software must be made available to editors and reviewers. We strongly encourage code deposition in a community repository (e.g. GitHub). See the Nature Portfolio guidelines for submitting code & software for further information.

## Data

Policy information about availability of data

All manuscripts must include a data availability statement. This statement should provide the following information, where applicable:

- Accession codes, unique identifiers, or web links for publicly available datasets
- A description of any restrictions on data availability
- For clinical datasets or third party data, please ensure that the statement adheres to our policy

Sequencing data has been uploaded to Gene Expression Omnibus (GEO GSE248237). Imaging data are available upon request. All other data is available in the main text or the extended data.

Published data have been retrieved from GEO (ATAC-Seq data, Palfy et al., 2020, GSE130944) and JASPAR database (2022 vertebrate core database, www.jaspar.elixir.no).

## Research involving human participants, their data, or biological material

Policy information about studies with human participants or human data. See also policy information about sex, gender (identity/presentation), and sexual orientation and race, ethnicity and racism.

| | |
|---|---|
| Reporting on sex and gender | N/A |
| Reporting on race, ethnicity, or other socially relevant groupings | N/A |
| Population characteristics | N/A |
| Recruitment | N/A |
| Ethics oversight | N/A |

Note that full information on the approval of the study protocol must also be provided in the manuscript.

# Field-specific reporting

Please select the one below that is the best fit for your research. If you are not sure, read the appropriate sections before making your selection.

☒ Life sciences ☐ Behavioural & social sciences ☐ Ecological, evolutionary & environmental sciences

For a reference copy of the document with all sections, see nature.com/documents/nr-reporting-summary-flat.pdf

# Life sciences study design

All studies must disclose on these points even when the disclosure is negative.

| | |
|---|---|
| Sample size | No statistical methods were used to predetermine sample size. Sample size was determined based on similar study in the field. Biological triplicates were performed for every experiment. Each biological experiment was obtained from different and independent embryo clutches. |
| Data exclusions | The mounting of embryos for live imaging exhibits common variability in quality, and poorly mounted samples were excluded from further analysis. All other data were included in the analysis. |
| Replication | All attempts at replication were successful. Experiments were repeated at least 3 times (biologically independent replicates) |
| Randomization | No animal or patient groups were used in our study, hence no randomization was required. |
| Blinding | Investigators were not blinded to group allocation. Where samples from different conditions were compared, the data acquisition was organized in a way to capture images from the overall sample in an unbiased manner, and analysis was carried out - where technically possible - using (semi)-automated analysis scripts, so that investigator bias is prevented during data acquisition and processing. |

# Reporting for specific materials, systems and methods

We require information from authors about some types of materials, experimental systems and methods used in many studies. Here, indicate whether each material, system or method listed is relevant to your study. If you are not sure if a list item applies to your research, read the appropriate section before selecting a response.

## Materials & experimental systems

| n/a | Involved in the study | |
|---|---|---|
| ☐ | ☒ | Antibodies |
| ☒ | ☐ | Eukaryotic cell lines |
| ☒ | ☐ | Palaeontology and archaeology |
| ☐ | ☒ | Animals and other organisms |
| ☒ | ☐ | Clinical data |
| ☒ | ☐ | Dual use research of concern |
| ☒ | ☐ | Plants |

## Methods

| n/a | Involved in the study | |
|---|---|---|
| ☒ | ☐ | ChIP-seq |
| ☒ | ☐ | Flow cytometry |
| ☒ | ☐ | MRI-based neuroimaging |

## Antibodies

| | |
|---|---|
| Antibodies used | Primary antibodies:<br>[1] Mouse IgM anti-Pol II CTD Ser2Phos (H5), monoclonal, ab24758 abcam<br>[2] Rabbit IgG anti-CDK9 (C12F7), monoclonal, Cell Signaling Technology<br><br>Secondary antibodies:<br>[3] Sheep IgG Anti-Digoxigenin-AP Fab fragments, 11093274910 Roche<br>[4] Donkey anti-rabbit IgG, conjugated with Alexa 488, A21206 Thermo Fisher<br>[5] Goat anti-mouse IgM, conjugated with Alexa 594, A21044 Thermo Fisher |
| Validation | Primary antibodies:<br>[1] This antibody clone (H5) was characterized as the main monoclonal antibody to recognize the serine 2 phosphorylation of the RNA polymerase II (Pol II) subunit 1 C-terminal domain heptad repeat (Patturajan, M. et al. 1998, Growth-related changes in phosphorylation of yeast RNA polymerase II, Journal of Biological Chemistry, 273(8), 4689–4694).The Pol II subunit 1 C-terminal domain heptad repeat is highly conserved in eukaryotes, and our own validation in zebrafish cells by immunofluorescence showed the expected distribution, the expected signal loss upon flavopiridol treatment, and overlap with ab193468 antibody in dual-color immunofluorescence.<br>[2] Supplier validation: IF on HeLa cells. Published validation: Western blot on zebrafish larvae (Matrone et al., 2015, Journal of Cell Science). Own validation: Positive comparison with live localization of cdk9-mNeonGreen overexpression.<br><br>Secondary antibodies:<br>[3-5] Selectivity confirmed by staining without primary antibodies or target hybridization probe. |

## Animals and other research organisms

Policy information about studies involving animals; ARRIVE guidelines recommended for reporting animal research, and Sex and Gender in Research

| | |
|---|---|
| Laboratory animals | The study used embryos of Wild Type (ABTL) and mir430-deletion mutant (Kuznetsova et al., 2023) zebrafish. No embryos older than 5 days were used, and in accordance with regulation these experiments are not formally considered animal experiments. Adult fish were maintained according to local husbandry regulation, which equally does not formally constitute animal experimentation. To obtain embryos, male and female fish were placed in a water tank with a separating net in the afternoon; the fish were placed together the following morning and embryos could be collected after few minutes of spontaneous mating. |
| Wild animals | No wild animals were used in the study. |
| Reporting on sex | We used embryos at a developmental stage at which the sex is not determined yet. However, zebrafish embryos develop into male and female adults with ruffly 50/50 frequency, which allows us to assume that the results presented in this study are not affected by any sex bias. |
| Field-collected samples | No field-collected samples were used in the study. |
| Ethics oversight | No ethical approval or guidance was required because our work is not considered animal experimentation by law. |

Note that full information on the approval of the study protocol must also be provided in the manuscript.
