# Supplementary figures and images for: Transcription bodies regulate gene expression by sequestering CDK9
Source: Nat Cell Biol. 2024 Apr 8;26(4):604–12. doi: 10.1038/s41556-024-01389-9 (PMC11021188; doi:10.1038/s41556-024-01389-9)

# Unprocessed images

Extended Data Figure 3c (left)

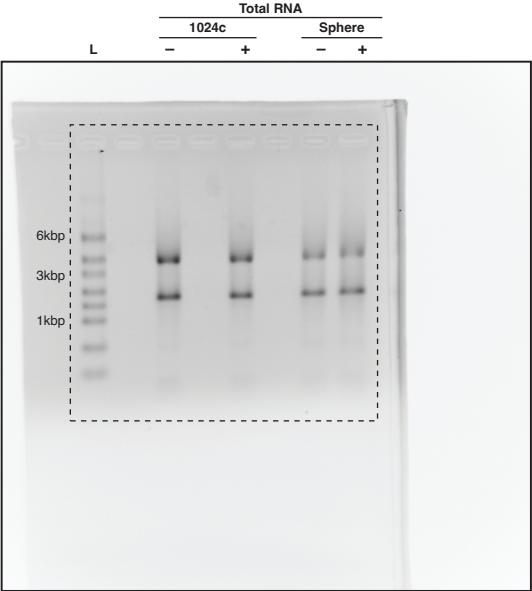

Extended Data Figure 3c (right)

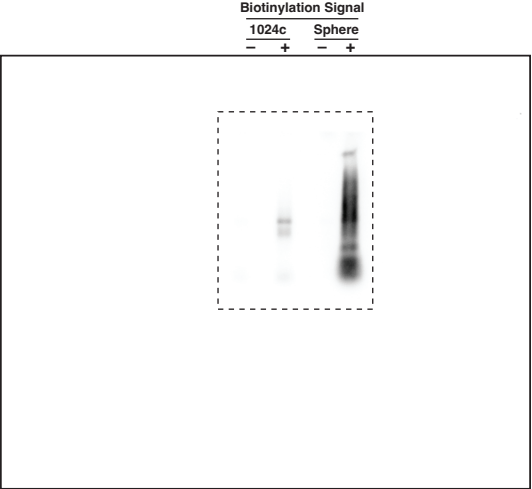

Supplement: Supplementary file 6 — Unprocessed images. [file 41556_2024_1389_MOESM6_ESM.pdf]
